# Supplementary material for: A modular organic neuromorphic spiking circuit for retina-inspired sensory coding and neurotransmitter-mediated neural pathways
Source: Nat Commun. 2024 Apr 3;15:2868. doi: 10.1038/s41467-024-47226-3 (PMC10991258; doi:10.1038/s41467-024-47226-3)
Supplement: Supplementary file 1 — Supplementary Information [file 41467_2024_47226_MOESM1_ESM.pdf]

Supplementary Information

A modular organic neuromorphic spiking circuit for retina-inspired sensory coding and neurotransmitter-mediated neural pathways

Giovanni Maria Matrone[1,2‡], Eveline R.W. van Doremaele[1‡], Abhijith Surendran[2], Zachary Laswick[2], Sophie Griggs[3], Gang Ye[4], Iain McCulloch[3,5], Francesca Santoro[6,7,8], Jonathan Rivnay[2], Yoeri van de Burgt[1]

‡These authors contributed equally to this work

[1]Microsystems, Institute for Complex Molecular Systems, Eindhoven University of Technology, 5612AJ, Eindhoven, The Netherlands.

[2]Department of Biomedical Engineering, Northwestern University, Evanston, IL 60208, USA.

[3]Department of Chemistry, Chemistry Research Laboratory, University of Oxford, Oxford, OX1 3TA, UK.

[4]Center for Biomedical Optics and Photonics (CBOP) & College of Physics and Optoelectronic Engineering, Key Laboratory of Optoelectronic Devices and Systems, Shenzhen University, Shenzhen 518060, P. R. China.

[5]King Abdullah University of Science and Technology (KAUST), KAUST Solar Center (KSC), Thuwal, 23955-6900, Saudi Arabia.

[6] Tissue Electronics, Istituto Italiano di Tecnologia, Naples, 80125, Italy.

[7] Institute of Biological Information Processing IBI-3 Bioelectronics, Forschungszentrum Juelich, 52428, Juelich, Germany.

[8] Neuroelectronic Interfaces, Faculty of Electrical Engineering and IT, RWTH Aachen, 52074, Aachen, Germany.

Correspondence to Giovanni Maria Matrone and Yoeri van de Burgt.

Supplementary Discussion 1: The organic spiking circuit and the benefits of the OECT-based design.

This section introduces the devices and materials that compose the spiking circuits (Supplementary Figure 1a,b). Before presenting the single devices characteristics, an overview is provided describing the state-of-the-art materials that are commonly used to design neuromorphic spiking circuits, highlighting the benefits of employing organic electrolyte materials/devices (OECT) comparing organic non-electrolyte (OFET) and inorganic technologies (Supplementary Table 1).
The circuital design of current spiking circuits is predominantly based on inorganic materials, particularly on silicon-based devices[1] (Supplementary Discussion 1, Supplementary Table 1). However, neuromorphic applications operating at the biointerface clearly favour organic materials[2], and specifically OECT-based neuromorphic circuits. Next to their relatively soft and flexible properties, high tunability and low operational voltage, organic mixed ion-electron conductors conduct both electrons and ions[3]. This mixed conduction allows organic materials to closely match the operating timescales of their biological counterparts[4], reduce interface impedance[5], and mimic ion-based biological functions such as neuronal ion-flux communication and neurotransmitter-receptor binding[2] (synaptic function), which are required to interact with biological tissues and to design adaptive biointerfaces[6]. Although showing desirable biocompatibility and flexibility properties, standard OFETs materials fail to translate (with the same sensibility and reliability of OECTs) the interaction of a complex bio-interfaced environment into a change of the neuromorphic circuit electrical characteristics.

Supplementary Table 1. List of the critical characteristics of artificial neurons and neuromorphic circuits based on different technologies. Critical neuromorphic characteristics comparing inorganic materials (grey), organic non-electrolyte materials (OFET-based, green), organic electrolyte materials (OECT-based, brown) neuromorphic systems.

	Circuit elements	Features	Footprint µm^2	Switching speed (single device)	Spike voltage amplitude [mV]	Operative Frequency [Hz]	Power or Energy consumption per spike	Operation in ionic environment	Connectivity to additional neurons
Si CMOS (based on integrate and fire or [7–11]	From 10 to 30+ transistors[9]	~ 20	993 µm^2	< 1ns	120[10] to 2300[9]	30[11]	< 100 fJ to 1nJ	no	yes
Mott-memristors[12–16]	2 memristors 1 capacitor 2 resistors	~ 20	100 µm^2 [13]	< 1ns	300[14]-3300[12]	10 to 10 kHz	~ 100 nW	no	yes

2D material Gaussian junction[17] and heterojunction [18,19]	>10transistors 3 resistors 3 capacitors	8 simulation	0.25 cm^2	-	450	< 1	250 nJ	no	no
OFET LIF[20–22]	3 resistors 3 transistors 2 capacitors	3	~ 10 mm^2	-	3000	200	20 µJ	no	yes
Complementary LIF[23,24]	5 transistors 1 capacitor	3	~ 10 mm^2	15 ms	4000	~ 2	0.5-40 nW	yes	no
Complementary LIF (this work)	5 transistors	3	~ 1 mm^2	120 ms	4000	~ 0.25	0.1 µJ	yes	yes

The devices and materials that compose the spiking circuits of this work (Supplementary Figure 1 a,b) are presented and the single device voltage and transfer characteristics are displayed (Supplementary Figure 3, Supplementary Figure 4, Supplementary Figure 5 and Supplementary Figure 6). Note that for all the experiments presented in the manuscript main text, the capacitor was disconnected from the circuit (see Fig.1) by omitting the electrolyte that connects the two sides of the capacitor.

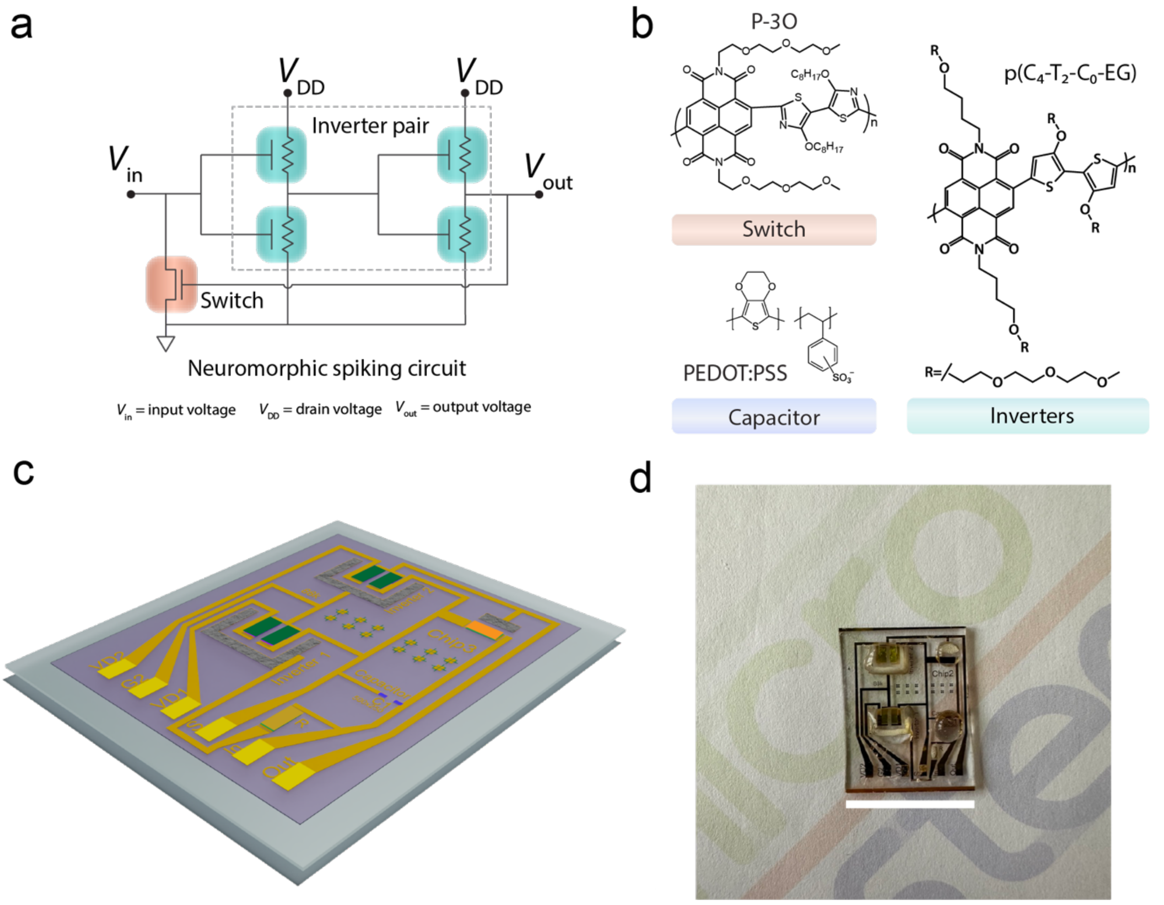

Supplementary Figure 1. The Neuromorphic spiking circuit chip. a) Schematic illustrating the neuromorphic spiking circuits connections. In orange the P-3O-based OECT used as an electronic switch, in blue the PEDOT:PSS-based capacitor and in green the p(C4-T2-C0-EG)-based OECTs composing the inverter pair. b) Materials used to fabricate the different electronic components and their molecular structure (same color code) c) Rendering of the circuit layout realized from the

photolithography dxf file. d) Photograph of the integrated circuit realized on a glass substrate, highlighting the total lateral footprint, scalebar = 15 mm.

The neuromorphic circuit was fabricated through standard photolithography (see the Methods section in the manuscript) on regular glass slides. Each integrated circuit occupies the space of a quarter of a 75 × 26 mm glass slide. The fabrication steps are detailed in Supplementary Figure 2. The circuit comprises four interdigitated arrays OECTs (green) for the two inverters, one interdigitated arrays OECT (orange) for the switch device and an in-plane two-plate capacitor. The interdigitated arrays have an electrode width of 10 µm and distance 5 µm, and comprises 22 finger pairs with length 2.2 mm. Supplementary Figure 1c shows a 3D rendering of the circuit, highlighting the single circuital elements with the same colour code.

The capacitor comprises two rectangular areas (500 × 250 µm) where PEDOT:PSS was spin coated. In Supplementary Figure 1d a photograph of the circuit substrate shows again the different components and how these are interfaced with the electrolyte (PBS).

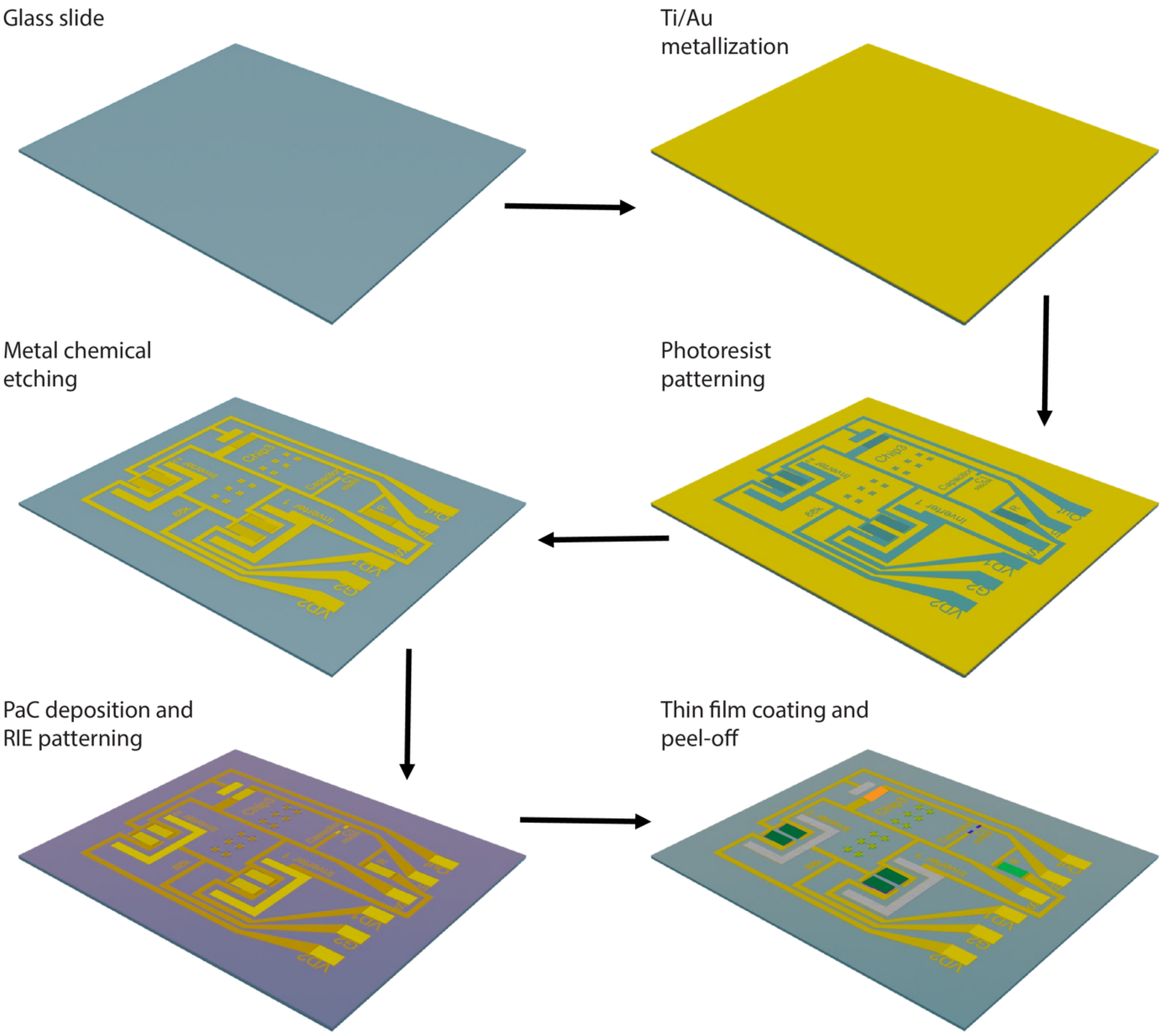

Supplementary Figure 2. Fabrication process for integrated spiking circuit. Thermal evaporation of 10/100 nm Ti/Au layers, photoresist (S1805) spin-coating and patterning, chemical etching of Au (Au-etchant) and Ti (HCl), double PaC deposition (insulating and sacrificial layers) and RIE patterning, polymer thin films spin-coating and peel off of the sacrificial PaC.

The spiking circuit capacitor (PEDOT: PSS OECT, blue) receives the external input in forms of voltage potential charging from 0 to 0.26 V (see Supplementary Figure 6). A 330 kOhm resistor is used to connect the capacitor to the *V*in. The capacitor is connected to the ground through a switch device (series connection through the channel terminal of this device, orange). In the experiments presented in the manuscript main text, the capacitor was disconnected from the circuit and consequently, the internal capacitance of the OECTs of the inverters and switch was used to integrate the input signal. Indeed, as reported in Supplementary Discussion 7, the overall system capacitance is dominated by the capacitance of the inverters and switch with a negligible contribution deriving from the capacitor element. This is the result of the scaling process which allowed us to accommodate all the elements on a quarter of a glass slide. The switch (P-3O OECT) opens and close the ground

connection of the capacitor depending on its resistance (Supplementary Figure 5). The polymer P-3O was synthesized following a previously reported protocol[25]. The switch device is in ON/OFF state depending on the voltage applied to its gate terminal and allows the capacitor to charge and discharge (Supplementary Figure 6). The inverter pair of the neuromorphic circuits is constituted by four identical p(C4-T2-C0-EG) OECTs (Supplementary Figure 1a, green). The polymer p(C_4-T2-C_0-EG) was synthesized following a previously reported protocol[26]. p(C4-T2-C0-EG) is an ambipolar material so the inverter of these OECTs can behave both as n-type and p-type transistors (Supplementary Figure 3). To the first inverter a V_{DD1}= 0.6V is applied, while on the second inverter V_{DD2} = 0.8V is applied. The V_{DD} unbalance was introduced to obtain a sharper inversion point at the second inverter (increasing de-facto the gain and the inversion range of the second capacitor) which is key to replicate neural spikes. When the capacitor (or the internal capacitance) charge reaches the inverters threshold (0.3 V), the first inverter characteristic voltage, due to the low V_{DD1}, moves from 0.6 to 0 V (Supplementary Figure 4, left axis). The second inverter, receiving as input voltage the output of the first inverter, displays a characteristic curve with an inversion point (0.26 V) with the voltage moving from 0 to 0.8 V (Supplementary Figure 4, red axis). The output terminal of the second inverter is connected to the gate terminal of the switch device so that the voltage inversion (0 to 0.8 V) of this system modulate the change of the state of the switch from OFF to ON (Supplementary Figure 5). When the switch is in the ON state the connection to the ground is closed and the capacitor discharges to 0 V, allowing a new cycle to start.

In Supplementary Figure 7 it is displayed a frequency modulation experiment with relevant parameters reported in Supplementary Table 2. In this case the capacitor (internal capacitance) was directly connected to an external voltage supplier and the voltage input was increased from 0.1 to 1.3 V. Through this experiment the threshold voltage for spikes initiation is identified. For values of the input voltage below 0.2V, the neuromorphic circuit does not spike: the capacitor charging time is slow and the circuit V_{OUT} reach an equilibrium state close to 0 V or some oscillations are shown. In the input voltage window 0.2 to 0.3 V the neuromorphic circuit shows irregular spikes. In this regime, due to the low input voltage, the total internal capacitance does not fully charge and then discharge in between each spike, so uncomplete spikes are recorded. Hence, it is impossible to extract a value for the spike frequency, since the spikes are irregularly spaced. Increasing the input voltage to 0.3 V, the neuromorphic circuit fires regularly spaced spikes. The extracted spike frequencies are reported in Supplementary Table 1, these are normalized respect to the starting frequency 0.1 Hz at 0.3 V, and the frequency increase with V_{IN} is reported in percentage (Supplementary Figure 7 and Supplementary Table 2). All these results are obtained from three different spiking circuit to check device reproducibility. Hence, the frequency values reported in the table are the average over three devices/experiments (standard deviation StDev is highlighted in Supplementary Table 1). Besides, still in Supplementary Table 2, the variation of the spike width is reported respect to the spike frequency. As the spike frequency change with the frequency, at each input voltage the full-width-at-

half-maximum is extracted from the previous series of data and reported in the table. Likewise, the amplitude of the spike in reported in the same table.

In Supplementary Figure 8 the spike frequency extracted from the previous data are plotted. With a linear regression (red line, $y = a + bx$) it is proved that the voltage/ frequency relation is sublinear (b = 0.13) in the voltage window examined (0.3 -1.3 V).

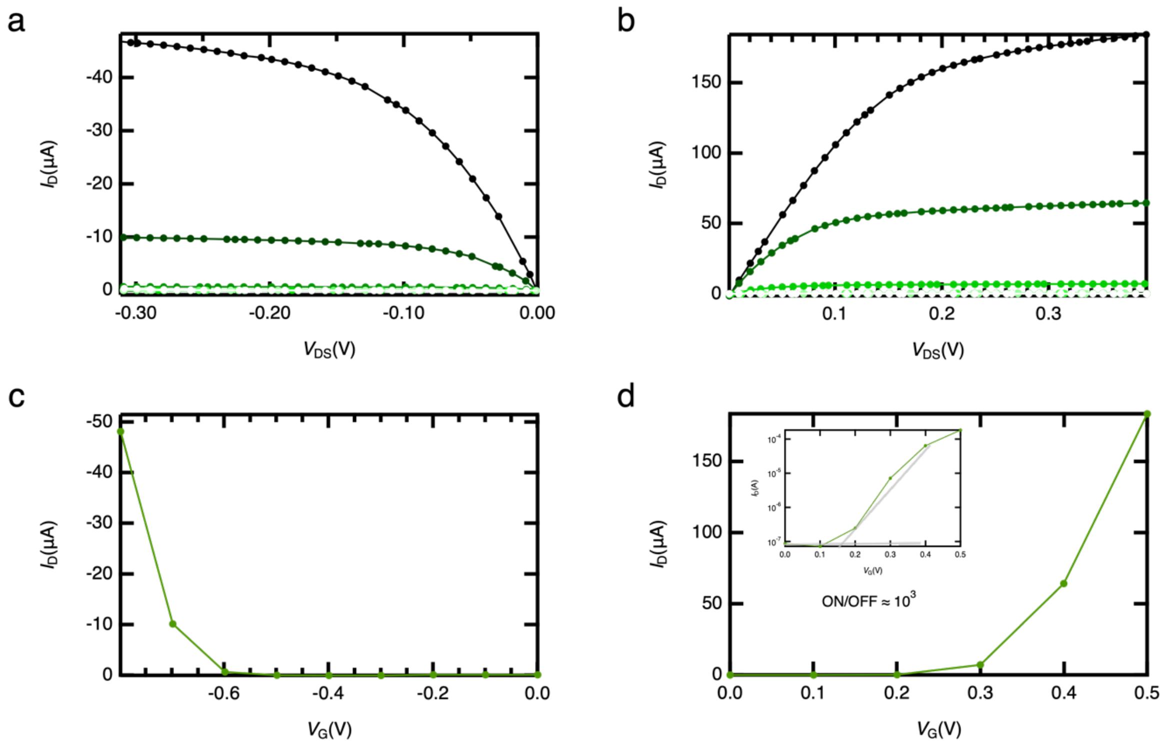

Supplementary Figure 3. Inverter materials electrical characteristics. p(C4-T2-C0-EG) transfer and output curves for both n-type and p-type behaviors. a) p-type output curve V_G from 0 to -0.8 V (step-size 0.1 V). b) n-type output curve V_G from 0 to 0.4 V (step-size 0.1V). c) p-type transfer curve at $V_{DS} = -0.2$ V. d) n-type transfer curve at $V_{DS} = 0.2$ V, in the inset the same curve on logarithmic scale highlight the ON/OFF ≈ 10^4 and the threshold voltage 0.16 V.

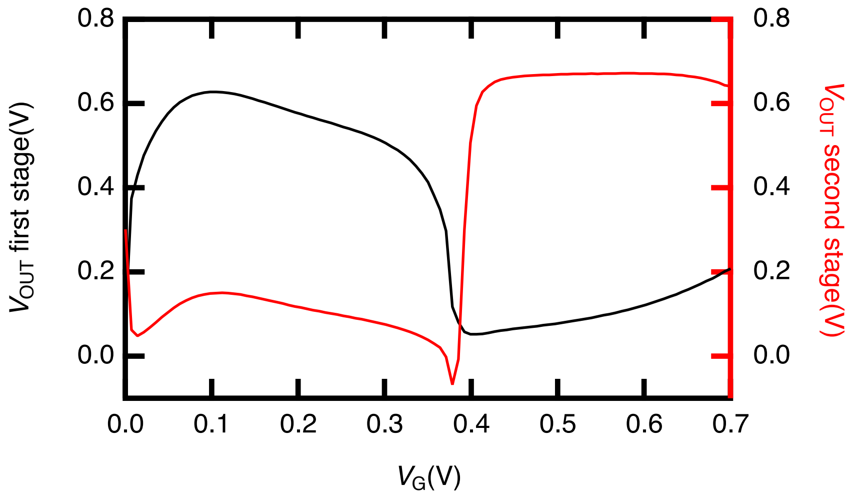

Supplementary Figure 4. Inverter characteristics. Inverter characteristics for both the first (single, black) inverter and the second (red) inverter comprising the inverter pair.

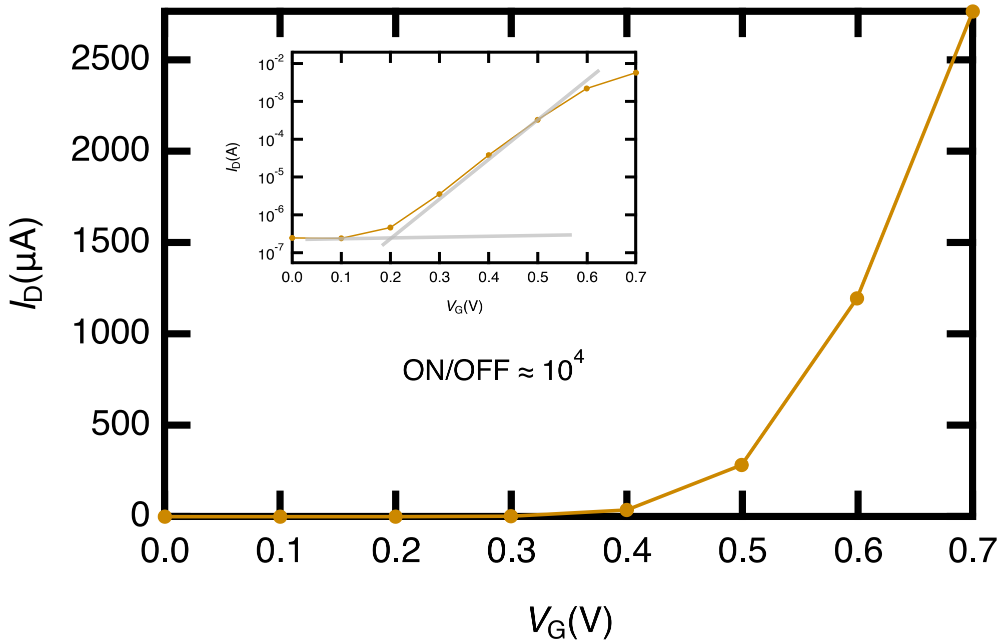

Supplementary Figure 5. Switch OECT transfer characteristic. P-3O transfer curve, in the inset the same curve on logarithmic scale highlight the ON/OFF ≈ 10^4 and the threshold voltage 0.2 V.

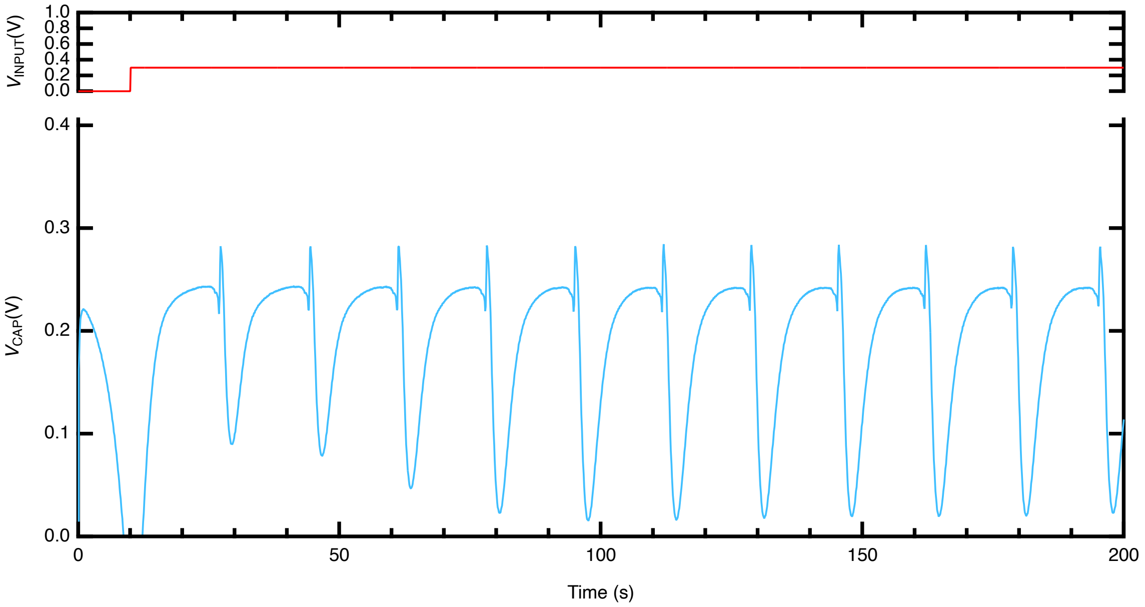

Supplementary Figure 6. Capacitor electrical characteristics. Capacitor characteristic (V_{Cap}) under a constant input voltage 0.3 V when connected to the neuromorphic circuit.

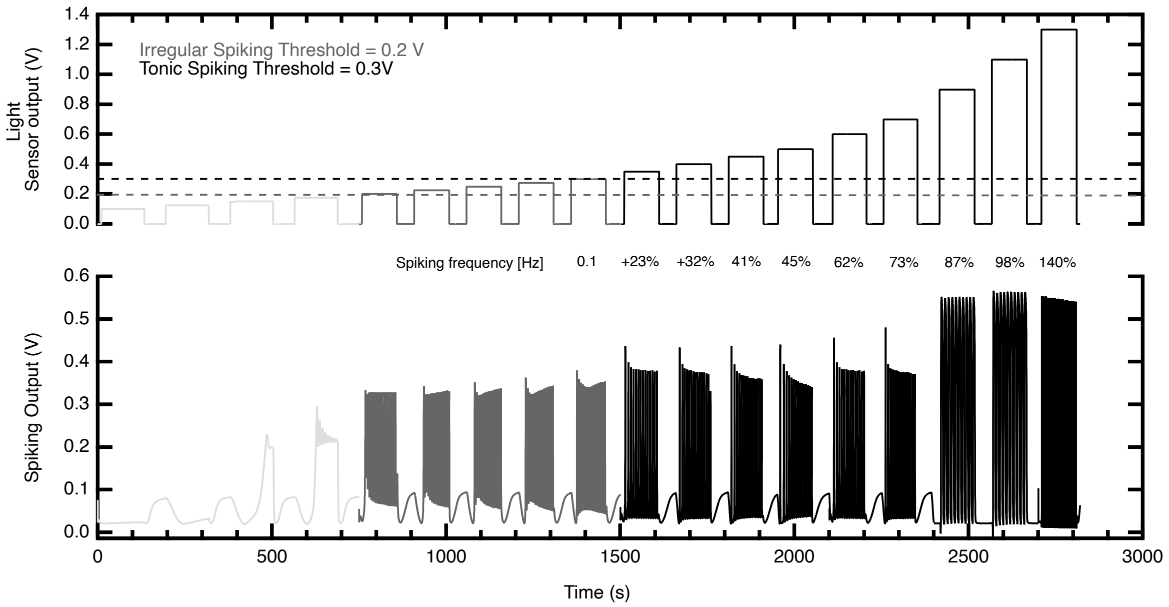

Supplementary Figure 7. Spiking circuit spikes patterns. The voltage spikes (spiking output) generated by the neuromorphic circuit responding to different voltages applied (light sensor output) by an external voltage supplier directly to the capacitor element. In between top and bottom panels, the spike frequency change (in percentage) corresponding to each spike pattern-voltage pair is reported.

Supplementary Table 2. Spike modulation of the spiking circuit. Spike modulation data as extracted from the trace in Supplementary Figure 7, comprising the input voltage, the time between each pair of spikes, the corresponding frequency

and the percentage of frequency change. The values reported are averaged over three devices/ experiment to check the neuromorphic circuit reproducibility.

Input voltage (V)	Spikes timing (s)	Frequency (Hz), stDev	Relative frequency increase (%)	Spike width (s)	Spike amplitude (V)
0.3	9.9	0,10 ± 0,05	-	2.1	0.35
0.35	8	0,13 ± 0,03	23,75	2.1	0.37
0.4	7.5	0,135± 0,01	32,00	2	0.37
0.45	7	0,14± 0,03	41,43	1.9	0.37
0.5	6.8	0,15± 0,04	45,59	1.85	0.37
0.6	6.1	0,16± 0,03	62,30	1.8	0.38
0.7	5.7	0,18± 0,01	73,68	1.75	0.38
0.9	5.3	0,185± 0,02	86,79	1.7	0.52
1.1	5	0,19± 0,04	98,00	1.6	0.52
1.3	4	0,25±0,01	147,50	1.5	0.51

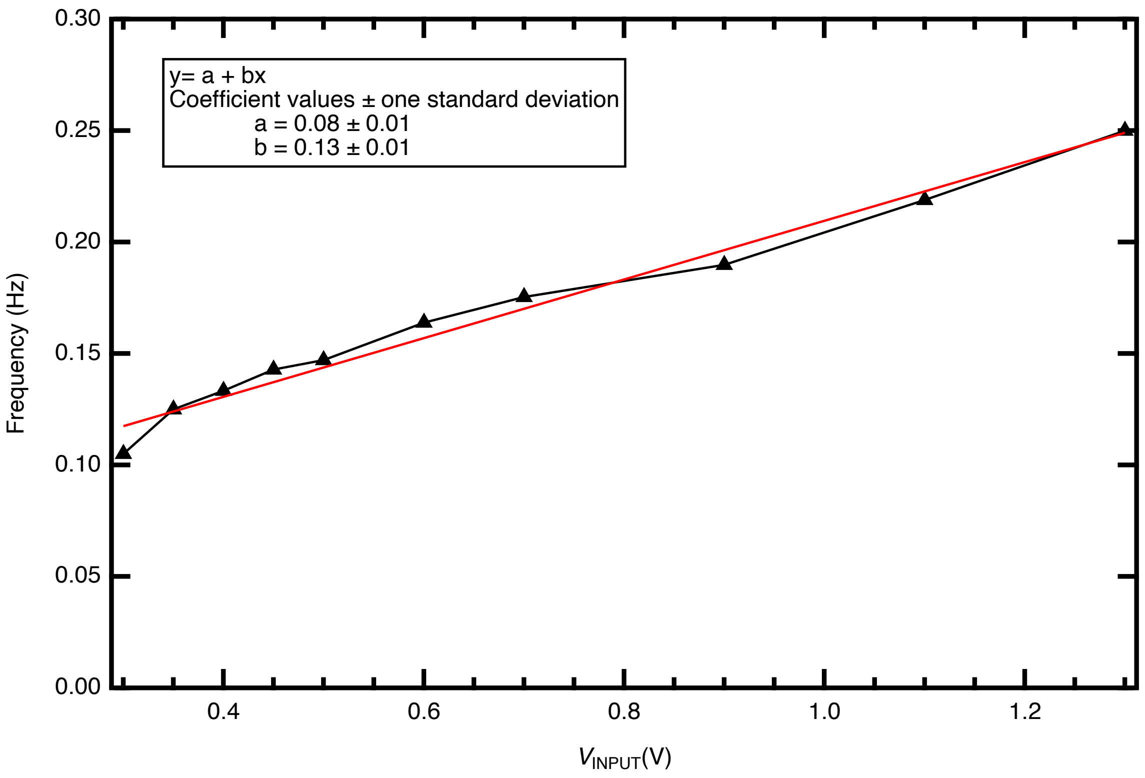

Supplementary Figure 8 The spike frequency modulation. Spike frequency modulation (depending on the V_{input}) as extracted from Supplementary Figure 7 traces. The plot shows that the relation is sublinear in the voltage region 0.3 – 1.3 V.

Supplementary Discussion 2: Sensory coding with light

Light sensor

In this section we describe the mechanisms of coupling of a commercially available light sensor to the neuromorphic spiking circuit to mimic the functions of an afferent neuron and perform sensory coding of light stimuli.

The light sensor we used is the DFRobot's Analog Ambient Light Sensor (DFR0026) with a voltage supply of 5 V (Supplementary Fig. 20). The light sensor was shielded from the ambient light except for a small opening on top of the sensor. With the use of an LED and a green 555 nm filter, we controlled the light stimuli delivering 0.01 mW/cm^2 (low light intensity) and 0.07 mW/cm^2 (high light intensity), which correspond to 11000 lux (ambient daylight) and 70000 lux (direct sunlight)[27]. As such, we replicated two common conditions experienced by the human retina: an ambient daylight close to the levels of an outdoor space, and the direct illumination encountered when facing the sun.

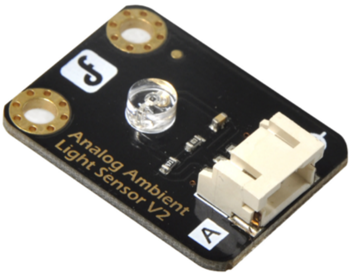

Light Sensor Output (V)	Optical power (mW/cm^2)	Optical power (lux)	Light Condition
0.3	0.01	11000	Ambient daylight
1.3	0.07	70000	Direct sunlight

Supplementary Figure 9. Ambient light sensor response. Analog ambient light sensor (supply voltage 5 V). In the table, the parameter extracted from the calibration of the sensor are reported with details on the optical power and the light conditions.

Hence, the light sensor was directly coupled to the input of the neuromorphic circuit as depicted in Supplementary Figure 10. As reported in Fig. 1 c and Supplementary Figure 11, two different light intensities have been employed to test the light sensory coding capability of the neuromorphic circuit and mimic the aforementioned biologically plausible conditions. Relevant parameters, extracted from the spiking pattern of Fig. 1c, are reported in Supplementary Table 3.

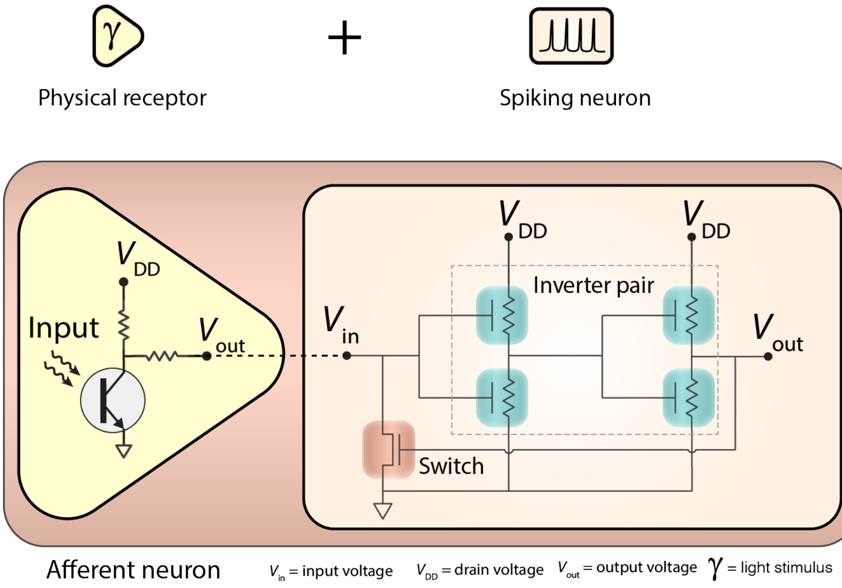

Supplementary Figure 10 Neuromorphic spiking circuit connected to the light sensor. Schematic illustrating the neuromorphic spiking circuits elements and the connection to the Light sensor to mimic the function of an afferent neuron.

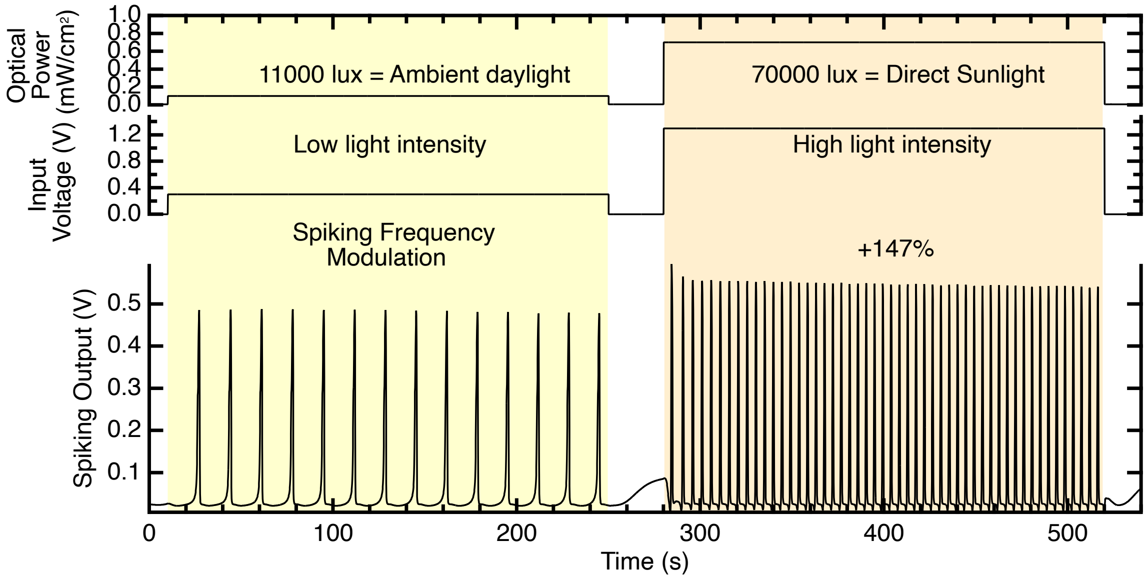

Supplementary Figure 11. From ligth intensity to input voltage and spiking output of the neuromorphic circuit. (corresponding to Fig.1c and here attached for improving the clarity of this dicussion). The spikes generated by the neuromorphic circuit responding to the light conditions (high and low light intensity) used in Fig.1 c.

Supplementary Table 3. Spike modulation data from different light conditions. Data extracted from the trace in Fig. 1 c and Supplementary Figure 11, comprising the light sensor output, the time between each pair of spikes, the corresponding frequency and the percentage of frequency change.

Light Sensor Output (V)	Spikes timing (s)	Frequency (Hz)	Relative frequency increase (%)

0.3	9.9	0,10	-
1.3	4	0,25	147,50

Calibration experiments are here reported (Supplementary Figure 11) showing a modulation of the spike frequency from 0.1 Hz to 0.25 Hz, as extracted in Supplementary Table 8, when moving from low light (0.3 V light sensor output) to high light intensity (1.2 V sensor output). Additionally, we investigate the neuron output response to voltage inputs that trigger the same number of spikes, in order to check the reproducibility and robustness of our encoding mechanism when stimuli of different duration are applied. In Supplementary Figure 12 the duration of light stimuli of different intensities is adjusted to ensure trains with only three spikes. As evident, the low light condition requires a duration time of 35 s to elicit a train of three spikes, while the high light condition three spikes train only requires a stimulus of 16 s. In both cases, train of spikes with amplitude, frequency and percentage modulation similar to the condition (long term stimuli) presented in Supplementary Figure 11 are recorded and listed in Supplementary Table 4. In summary, the output of the spiking circuit does not depend on the duration of the stimulus, if the stimulation time, depending on the input voltage, allows to trigger the spikes activity.

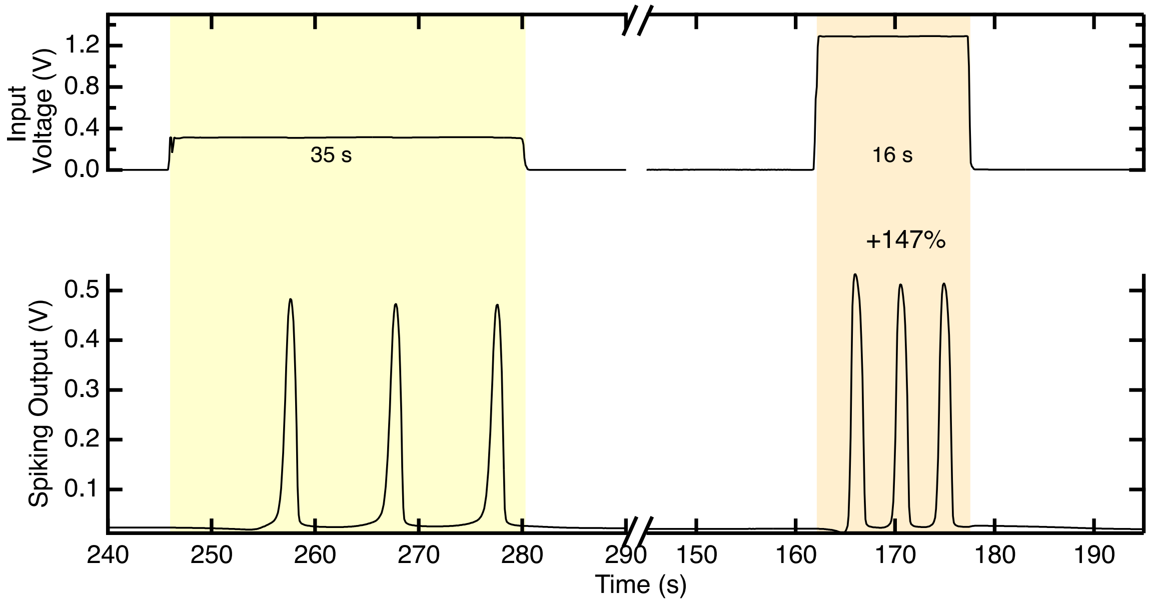

Supplementary Figure 12. Three-spikes output of the neuromorphic circuit. The output generated by the neuromorphic circuit responding to the light conditions (low and high light intensity) whose duration triggers a train of three spikes.

Supplementary Table 4. Spike modulation data for short time light stimuli. Spikes data for short time light stimuli (triggering three spikes) as extracted from the trace in Supplementary Figure 12, comprising the light sensor output, the time between each pair of spikes, the corresponding frequency and the percentage of frequency change.

Light Sensor Output (V)	Spikes timing (s)	Frequency (Hz)	Relative frequency increase (%)
0.3	10	0,10	-
1.3	4.1	0,245	145

Supplementary Discussion 3: biohybrid synapse to emulate the release of neurotransmitters in neuromorphic systems.

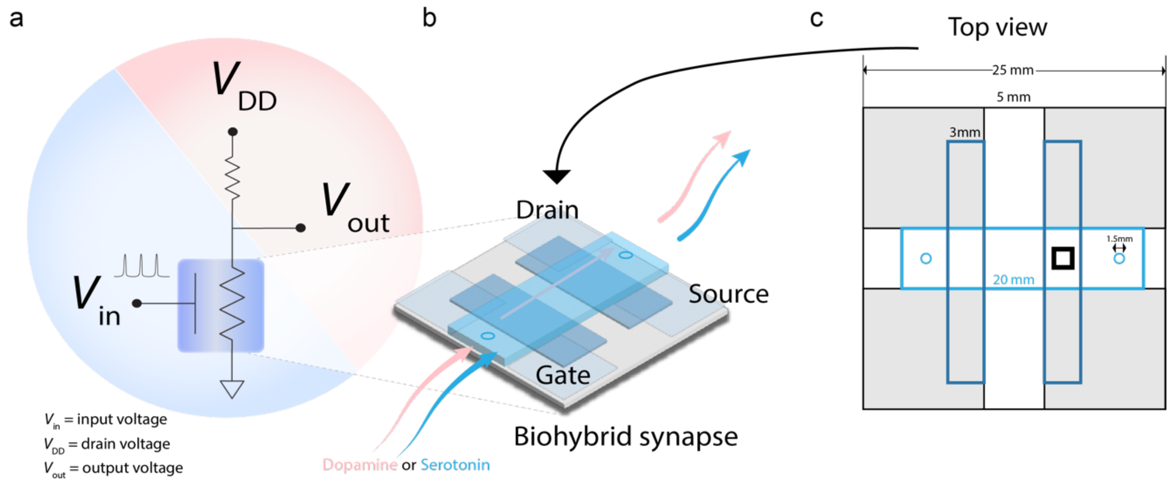

Supplementary Figure 13. The biohybrid synapse and the voltage divider. a) The connections of the synaptic modulator and its components and b) close-up of the bio-hybrid synapse component. c) The top view of the bio-hybrid synapse.

In Supplementary Figure 13a,b the neurotransmitter-mediated neuromorphic device included in the synaptic modulator (presented in the manuscript), the so-called bio-hybrid synapse, is depicted,

showing the Gate, Source, Drain ITO electrodes. The design of this device is based on a previous work[28].

In the same panel, the fluidic module is in pale blue and shows the direction of the flow. Supplementary Figure 13c panel depicts top view of the transparent glass where the grey areas represent the ITO electrode, the dark blue rectangles are the PEDOT:PSS films used as gate and channel of the device, the light blue area corresponds to overlap of the microfluidic module with the substrate. As such, the channel and gate PEDOT:PSS areas, the active areas in contact with the electrolyte, both measure 3 × 5 mm. The circles indicates the position of the inlet hole of the microfluidic module.

The thickness of the PEDOT:PSS films was determined in a dry state after testing with a DEKTAK profilometer (average over three film, 120-130 nm).

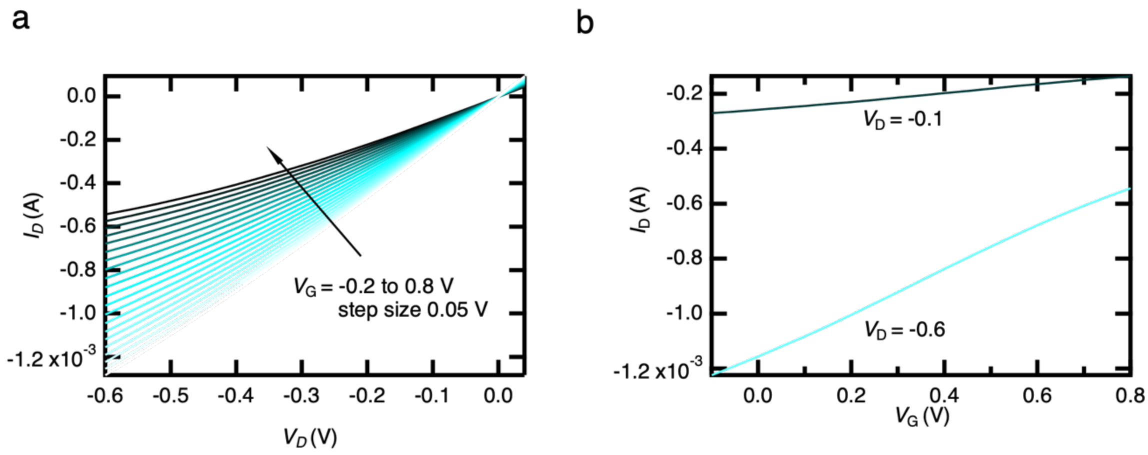

Supplementary Figure 14. Biohybrid synapse electrical characteristics. a) Output characteristic of the biohybrid synapse operated as an OECT with V_G varying from -0.2 to 0.8 V. b) Transfer characteristic of the device operated as an OECT with drain voltages V_D -0.1 and -0.6 V.

The device work as a conventional organic electrochemical transistor (OECT) based on the material poly(3,4-ethylene- dioxythiophene):polystyrene sulfonate (PEDOT:PSS), with a gate constituted by the same material. Hence, the device operates in depletion mode and the application of a voltage bias at the gate electrode causes a de-doping of the film due to cations penetrating its bulk (Supplementary Fig. 1 d,e).[29] This mechanism is reversed upon removal of the voltage bias. Employing voltage pulses as inputs, the device can operate as an ENODE achieving a volatile control of the conductivity of the organic mixed ionic/electronic conductor when no neurotransmitters are present. In this condition, a reversible conductance modulation is achieved (Fig. 1b, black dotted traces).

However, in presence of a neurotransmitter, the PEDOT:PSS channel conductance decreases in a non-reversible way due to the de-doping process (Fig. 1b**,** red and blue solid traces)[30]. This mechanism is strongly influenced by protons and electrons produced during the oxidation reaction thus depending on the type of neurotransmitter and its concentration in the solution as previously shown.[30]

In Supplementary Figure 15 the bio-hybrid synapse conductance modulation is reported in case dopamine (left) and serotonin (right) are employed. In this experiment the input voltage applied to the bio-hybrid gate consist of three periodic voltage pulses of 300 mV (dopamine) and 400 mV (serotonin) with time width 3 s and delay of 10 s[28]. The conductance G_D depends on the concentration of neurotransmitter and on the number of voltage pulses applied (in these graphs only three). Increasing the number of input gate pulses, the dynamic range of the device saturates (as seen in Fig.1 c for DA) and the conductance modulation per pulse decreases.

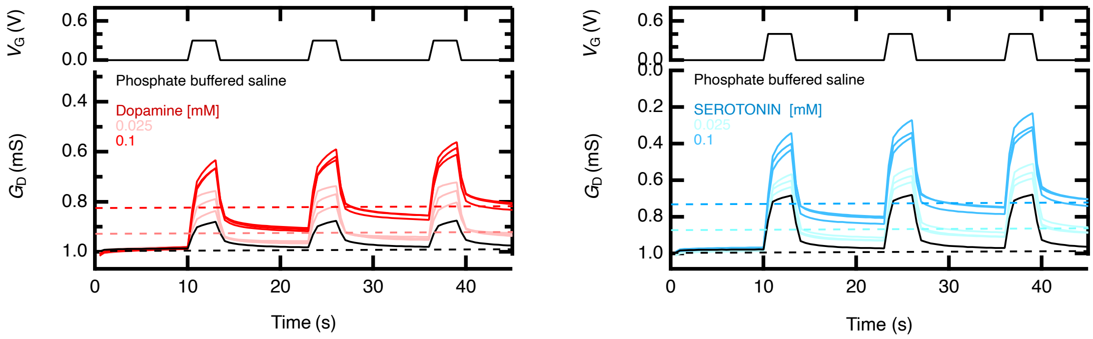

Supplementary Figure 15. Biohybrid synapse characteristic in presence of neurotransmitters. Biohybrid synapse characteristics conductance modulations with (colors) and without (black) dopamine (DA) and serotonin (5-HT) neurotransmitters.

Form the previous work, we present an overview on the mechanism of conductance modulation in such devices. The conductance modulation of the bio-hybrid synapse can be explained by the two possible reactions that can occur at the PEDOT:PSS electrodes. The first reaction is the two- electron oxidation of dopamine or serotonin as described:

$$DA \rightarrow DQ + 2e^{-} + 2e^{+} \quad (1)$$

$$5HT \rightarrow 5HTQ + 2e^{-} + 2e^{+} \quad (2)$$

Where DA is dopamine, DQ is dopamine *o*-quinone[31] and 5HTQ is the serotonin quinone[32,33]. The Eq. 1 and 2 reactions result in the de-doping of PEDOT:PSS (the decrease of its conductance) as shown in Supplementary Fig. 13 and given by the following equation.

$$PEDOT^{+}: PSS^{-} + e^{-} + Cat^{+} \rightarrow PEDOT^{0} + PSS^{-}: Cat^{+} \quad (3)$$

Where the electron reduces $PEDOT^{+}$, eliminating a hole and leading to a decrease in conductivity, and the proton or other cationic species (Cat^{+}) compensates the negative charge of the sulfonate group on PSS. The previous reactions lead to a decrease in the channel conductance bio-hybrid synapse.

Our neuromorphic system demonstrates for the first time an artificial network of neurons connected through chemical synapses (biohybrid synapses) which work to adjust the synaptic weights depending on the neurotransmitter environment and regulate in cascade the spike frequency along a neural pathway.

This system has been developed with the scope of future bio-integration. In this context, a previous work by Keene et al. demonstrated the use of the bio-hybrid synapse (with a device working on the same principles of our synapse) as a platform interfaced with PC-12 cells. It allowed the transduction of chemical signals of these cells (released dopamine) into permanent conductance modulations of the organic neuromorphic device's channel terminal (chemical to electrical signal transduction)[30]. In the more recent work of Matrone et al., to investigate the interaction of electroactive neurotransmitters (dopamine and serotonin) without interfering agents (additional molecules expressed by the cell models), the sending chemical signal (neurotransmitter release) was substituted by a controllable microfluidic module[28]. In the same context, in our work the chemical messengers' activity is emulated by the activation of the microfluidic module. However, even though this design choice introduced a gap between biological and artificial synapses for a practical control of neurotransmitters release, the proposed system has been clearly developed on the same design rules used by previous bio-interfaced devices[30]. Hence, the biohybrid synapse can be interfaced with cells models that express electroactive neurotransmitters such as dopamine and serotonin[34]. In this case, the biohybrid synapse conductance state depends on the electrolyte environment (which is controlled by the interfaced cells). Hence, the spiking circuit connected to this synapse will show frequency adaptation depending on the cell activity (neurotransmitter expression).

For example, a sending neuron signal can be used to trigger the release of neurotransmitters (electrical stimulation) from the interfaced cells, and promote the oxidation of these molecules to control the output of the synaptic modulator[35], eliciting in cascade an increase of the receiving neuron spikes frequency.

On the other hand, on a still artificial design level without the use of bio-interfaced tissues, alternative approaches to locally deliver neurotransmitters and replicate the synaptic machinery have been also proved[36,37].

Supplementary Discussion 4: Synaptic modulator with dopamine and serotonin for frequency tuning and signal transmission.

In this section the strategy to employ the bio-hybrid synapse to modulate the spike frequency of the neuromorphic circuit is presented. The bio-hybrid synapse from previous section (Supplementary Figure 13) is included in a voltage divider configuration (Supplementary Figure 16a) connected in series with a 1 kOhm resistor, hereinafter referred as synaptic modulator (Synmod). The V_{OUT} of this voltage

divider depends on the ratio of the resistance of the two elements comprising the circuit according to the formula:

$$V_{\mathrm{OUT}} = \frac{R_{\mathrm{O.S.}}}{(R_{\mathrm{O.S.}} + R_{1\mathrm{kOhm}})} \quad (4)$$

We also note that the V_{OUT} is controlled by the value of V_{DD} applied, as explained at the end of this section. In the above formula we assume the bio-hybrid synapse to be a tuneable resistor. Indeed, the resistance of the PEDOT:PSS-based can be tuned by applying a gate potential > 0 V , i.e. switching off the OECT. In Supplementary Figure 13, the conductance of the bio-hybrid synapse was modulated applying periodic voltage pulses (generated by an external voltage supply) on the gate of the device, exploiting the oxidation of dopamine and serotonin. However, in this work the Synmod mimic the function of a biological synapse, the V_{OUT} is connected to neuromorphic spiking circuit, replicating the functions of an interneuron (Fig.1 d and Supplementary Figure 17), while the bio-hybrid synapse receives the spikes from an afferent neuron. Supplementary Figure 22 depicts the laboratory setup that allowed the measurements. Each neuron is connected to the Arkeo system with the clips depicted in the photograph. The artificial synapses are connected to Arkeo and so to the neurons via probes. The two syringe pumps were used to control the presence of DA and 5-HT in the bio-hybrid synapses. In this configuration, in the neutral electrolyte PBS (no neurotransmitter in solution) the Afferent neuron spikes applied to the gate of the biohybrid synapse enable a reversible modulation of its resistance. Indeed, the V_{OUT} of the voltage divider does not change (Supplementary Figure 18). When DA or 5-HT are introduced in solution, using a fluidic module as depicted in Supplementary Figure 22, the oxidation of these molecules leads to an increase of the resistance of the bio-hybrid synapse that depends on the numbers of pulses applied and on the concentration of the neurotransmitters. Hence, the voltage divider output increases. This is used as input voltage to charge the capacitor (internal capacitance) of the spiking circuit. In short, the spike frequency is modulated (increases) depending on the type of neurotransmitter used and on the pulsing time.

Supplementary Figure 18 and Supplementary Figure 19 report the spiking output traces (Fig. 1d) that have been used to extract the evolution of the frequency modulation (Supplementary Table 6 and Supplementary Table 7), under high light and low light conditions, respectively, when two different DA concentrations (0.025 mM and 0.1 mM) are introduced via the fluidic module (black arrows in the Supplementary Figure 18 and Supplementary Figure 19).

As for DA in Fig.1 d and Supplementary Figure 18, the same mechanism has been replicated with the use of two different concentrations of 5-HT (0.01 mM and 0.05 mM). Indeed, Supplementary Figure 20 is the replica of Fig.1 d and Supplementary Figure 18 (high light condition) with the use of 5-HT while Supplementary Figure 21 is the replica of Supplementary Figure 19. The results of the Synmod change and frequency with these neurotransmitter concentrations are reported in Supplementary Supplementary Table 7 and Supplementary Supplementary Table 8.

For the experiments reported in Fig. 1 d of the manuscript the V_{DD} of the synaptic modulator is set to 0.85 V. Generally, by tuning the V_{DD}, the output of the Synmod can be adjusted to match the desired application. As anticipated, in normal (spiking) condition, as from Supplementary Figure 18 and Supplementary Figure 21, the V_{DD} is set to 0.85V corresponding to a V_{OUT} close or above 0.3V. This value of V_{DD} has been selected in order to investigate the effects of DA and 5-HT on the V_{OUT} while using this output to operate a connected neuron, monitoring in cascade the variations of the spike frequency. As from Supplementary Discussion 1, a V_{OUT} close to 0.3 V allows the connected neuron to operate in the spiking regime. In Fig.2 of the manuscript, two synaptic modulators are used to connect two neurons demonstrating the activation of a neural pathway. In this case, both neurons start from a non-spiking regime $V_{OUT} < 0.2$ V. For the the experiments reported in Fig. 2 of the manuscript the V_{DD} of the DA synaptic modulator is set to 0.65V and the V_{DD} of the 5-HT synaptic modulator is set to 0.7 V. The former V_{DD} values correspond to values of V_{OUT} of 0.1V and 0.14 V for the DA and 5-HT neuron, respectively. Hence, the values of V_{DD} have been adjusted to keep the neuron in a silent state $V_{OUT} < 0.2$ V, allowing the specific change in V_{OUT} induced by the biological cues (DA and 5-HT) to be a trigger for the spikes activation (i.e., increasing the V_{OUT} to 0.2 V). We report in Supplementary Figure 16 the variations of the V_{OUT} of the synaptic modulator with the V_{DD} values.

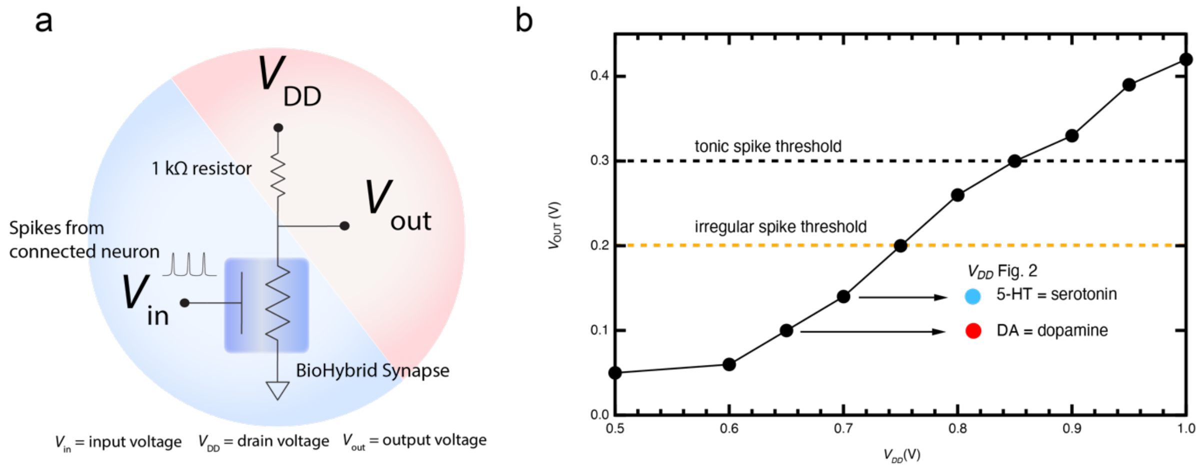

Supplementary Figure 16. Voltage divider configuration (synaptic modulator) for exploiting the biohybrid synapse as a chemical receptor. a) Schematic of the voltage divider, b) *V*out of the voltage divider respect highlighting the dopamine (DA) and serotonin (5-HT) levels.

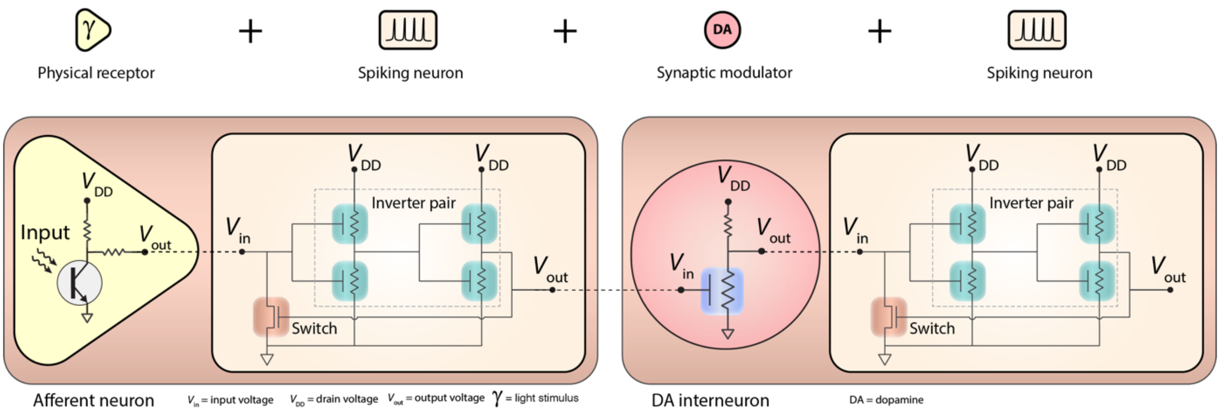

Supplementary Figure 17. Light sensor to spiking neuron to neurotransmitter spiking neuron connection. Schematic illustrating the neuromorphic spiking circuits elements and the connection to the Synaptic modulator to mimic the function of an interneuron.

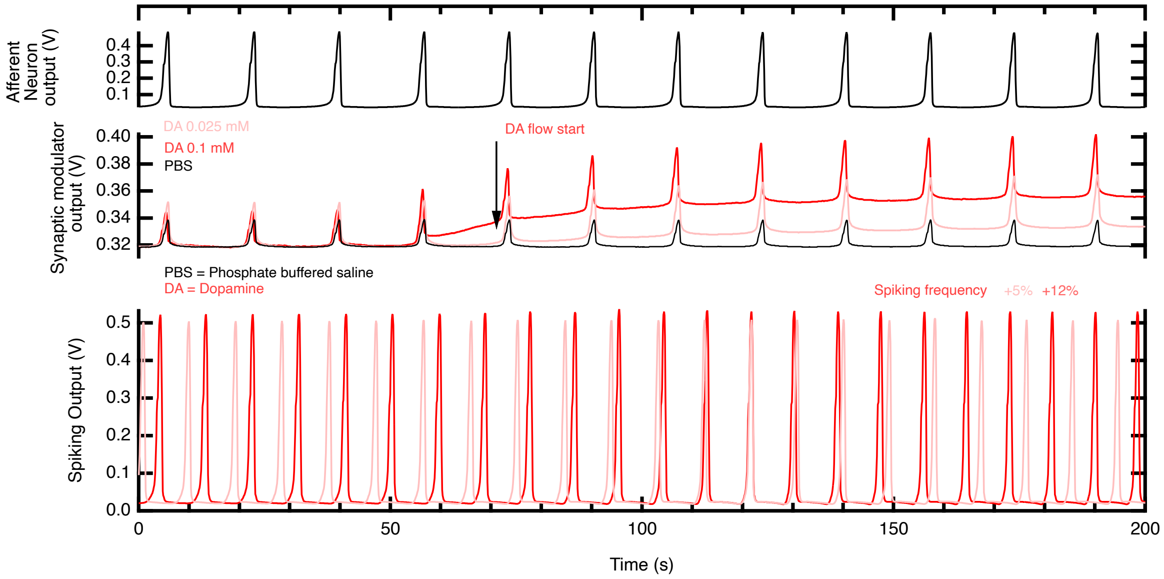

Supplementary Figure 18. Neuromodulation with dopamine at low high intensity. This figure correspond to Fig.1d, with the raw versions of the synpatic modulator output curves showing the spikes with solid lines (here attached for improving the clarity of this dicussion). Neuromodulation with dopamine using the Synaptic modulator showing the modulation of the Interneuron spike frequency depending on the dopamine concentration. The input signal to the Synaptic modulator is a train of spikes from the afferent neuron under high light intensity. The frequency modulation is in percentage (dark red for high DA concentration and light red for low DA concentration) with respect to the spike frequency of PBS, black (0.10 Hz).

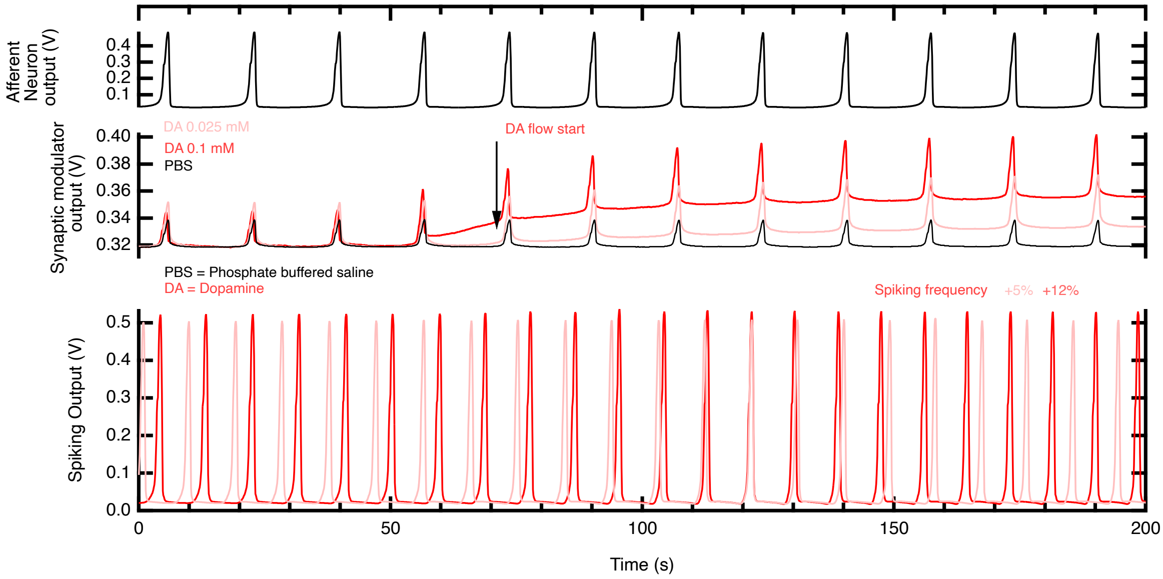

Supplementary Figure 19. Neuromodulation with dopamine at low light intensity. Neuromodulation with dopamine using the Synaptic modulator showing the modulation of the Interneuron spike frequency depending on the dopamine concentration. The input signal to the Synaptic modulator is a train of spikes from the afferent neuron under low light intensity. The frequency modulation is in percentage (dark red for high DA concentration and light red for low DA concentration) with respect to the spike frequency of PBS, black (0.10 Hz).

Supplementary Table 5. Spike modulation data for different dopamine concentrations at high light intensity. Spike modulation data as extracted from the trace in Fig. 1 d and Supplementary Figure 18 for Dopamine (DA) 0.1 mM and 0.025 mM (high light intensity as afferent neuron input), comprising the time, the voltage divider (synaptic modulator) output, the time between each pair of spikes, the corresponding frequency and the percentage of frequency change.

	Time (s)	Voltage Divider Output(V)	Spikes timing (s)	Frequency (Hz)	Relative frequency increase (%)
DA 0.1mM	0	0.32	9.9	0.1	-
	200	0.40	7.7	0.13	28%
DA 0.025mM	0	0.32	9.9	0.1	-
	200	0.38	7.85	0.12	26%

Supplementary Table 6. Spike modulation data for different dopamine concentrations at low light intensity. Spike modulation data as extracted from the trace in Supplementary Figure 19 for Dopamine 0.1 mM and 0.025 mM (low light

intensity as afferent neuron input), comprising the time, the voltage divider (synaptic modulator) output, the time between each pair of spikes, the corresponding frequency and the percentage of frequency change.

	Time (s)	Voltage Divider Output(V)	Spikes timing (s)	Frequency (Hz)	Relative frequency increase (%)
DA 0.1mM	0	0.32	9.9	0.10	-
	200	0.34	8.8	0.11	12%
DA 0.025mM	0	0.32	9.9	0.1	-
	200	0.34	7.85	0.105	5%

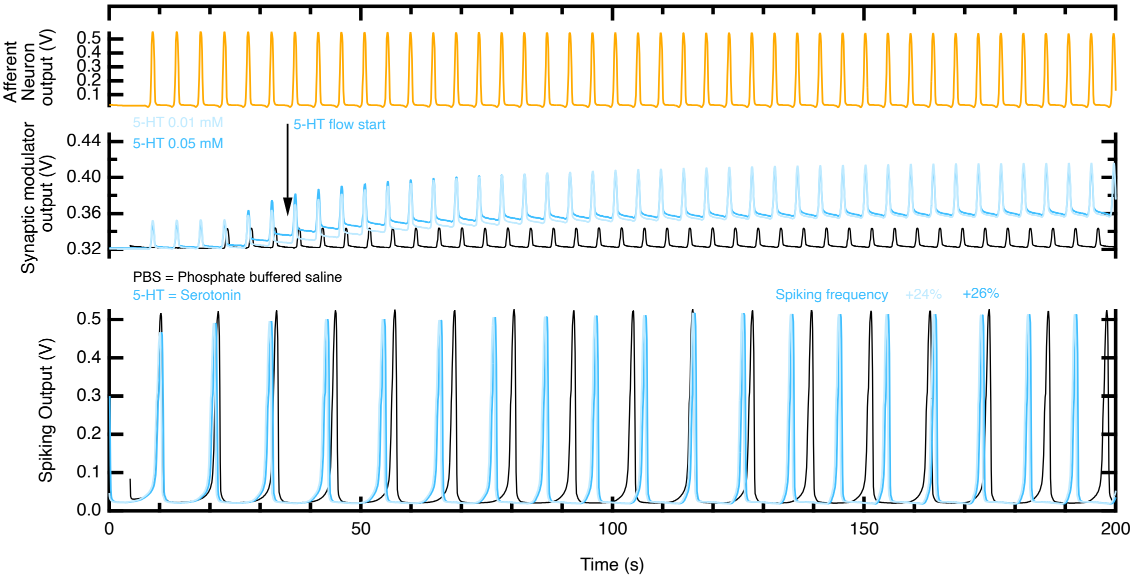

Supplementary Figure 20. Neuromodulation with serotonin at low high light intensity. Neuromodulation with serotonin using the Synaptic modulator showing the modulation of the Interneuron spike frequency depending on the serotonin concentration. The input signal to the Synaptic modulator is a train of spikes from the afferent neuron under high light intensity. The frequency modulation is in percentage (dark blue for high 5-HT concentration and light blue for low 5-HT concentration) with respect to the spike frequency of PBS, black (0.10 Hz).

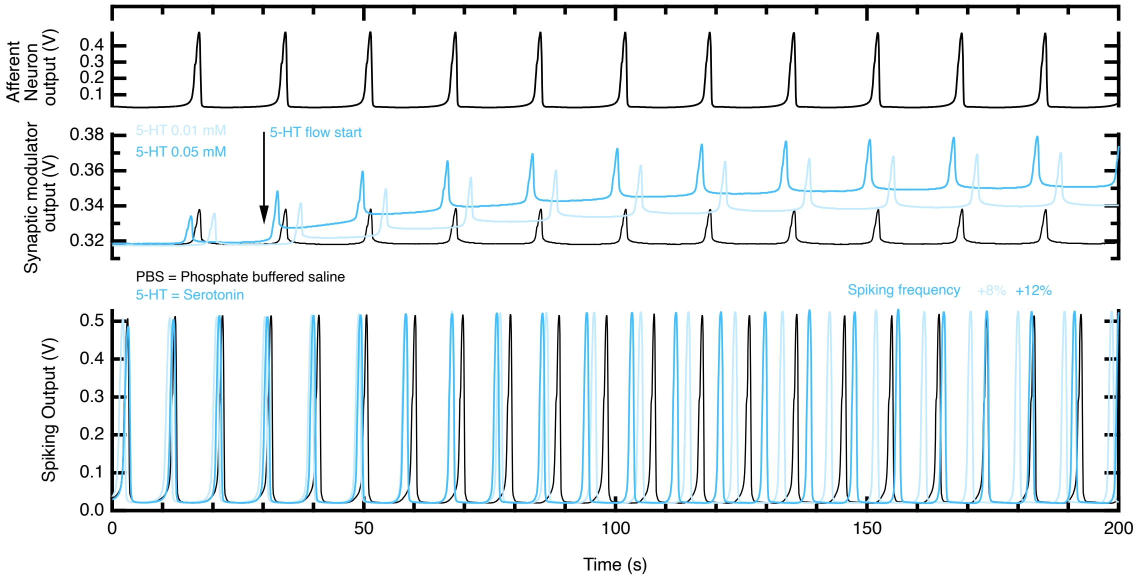

Supplementary Figure 21. Neuromodulation with serotonin at low low light intensity. Neuromodulation with serotonin using the Synaptic modulator showing the modulation of the Interneuron spike frequency depending on the serotonin concentration. The input signal to the Synaptic modulator is a train of spikes from the afferent neuron under low light intensity. The frequency modulation is in percentage (dark blue for high 5-HT concentration and light blue for low 5-HT concentration) with respect to the spike frequency of PBS, black (0.10 Hz).

Supplementary Table 7. Spike modulation data for different serotonin concentrations at high light intensity. Spike modulation data as extracted from the trace in Supplementary Figure 20 for 5-HT 0.05 mM and DA 0.01mM (high light intensity as afferent neuron input), comprising the time, the voltage divider (synaptic modulator) output, the time between each pair of spikes, the corresponding frequency and the percentage of frequency change.

	Time (s)	Voltage Divider Output(V)	Spikes timing (s)	Frequency (Hz)	Relative frequency increase (%)
5-HT 0.05mM	0	0.32	9.9	0.10	-
	200	0.345	8.8	0.115	12%
5-HT 0.01mM	0	0.32	9.9	0.1	-
	200	0.33	9.2	0.11	8%

Supplementary Table 8. Spike modulation data for different serotonin concentrations at low light intensity Spike modulation data as extracted from the trace in Supplementary Figure 21 for 5-HT 0.05 mM and DA 0.01 mM (low light

intensity as afferent neuron input), comprising the time, the voltage divider (synaptic modulator) output, the time between each pair of spikes, the corresponding frequency and the percentage of frequency change.

	Time (s)	Voltage Divider Output (V)	Spikes timing (s)	Frequency (Hz)	Relative frequency increase (%)
5-HT 0.05 mM	0	0.32	9.9	0.10	-
	200	0.37	7.85	0.13	26%
5-HT 0.01 mM	0	0.32	9.9	0.1	-
	200	0.37	8	0.125	24%

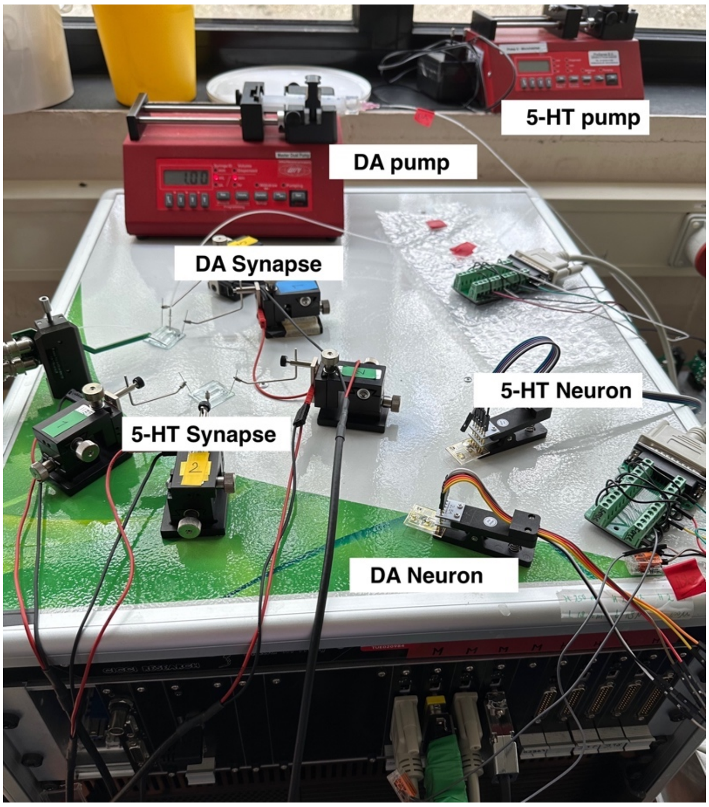

Supplementary Figure 22. Spiking neurons to voltage dividers setup. Photograph of the setup used in the laboratory to connect each neuron (DA and 5-HT) to its synaptic modulator (DA and 5-HT) synapse and activate the fluidic module to control the expression of the neurotransmitters.

Supplementary Discussion 5: Cascade Neural Pathway and its biological relevance.

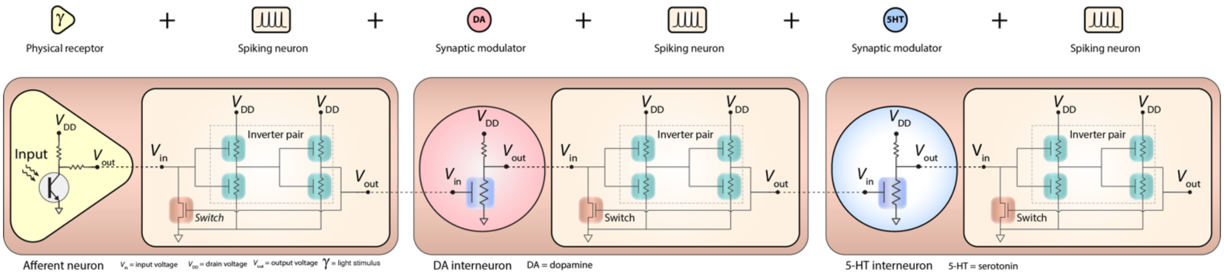

Supplementary Figure 23. Light sensor to spiking neuron to neurotransmitter spiking neurons connection. Schematic illustrating the neuromorphic spiking circuits elements and the connection to the Light sensor to mimic the function of an afferent neuron. The output of the Afferent neuron (spikes) is coupled to the gate of the bio-hybrid synapse of a synaptic modulator which is connected to another spiking circuit, thus replicating the functions of an Interneuron (DA neuron). From this DA-Interneuron a similar connection is established to a 5-HT Interneuron.

In order to perform the experiment of Fig.3, two spiking circuits (Interneurons) were connected in series using two bio-hybrid synapses in Synmod configuration to influence the spike frequency of the corresponding neuron. The first Interneuron (DA-Interneuron) receives the input (spikes) on its bio-hybrid synapse mediated by DA from an Afferent neuron (Supplementary Figure 23). Supplementary Figure 22 depicts the laboratory setup that allowed the measurements. Each neuron is connected to the Arkeo system with the clips depicted in the photograph. The artificial synapses are connected to Arkeo and so to the neurons via probes. The two syringe pumps were used to control the presence of DA and 5-HT in the artificial synapse. At the beginning of the experiment, the artificial synapses operate with PBS, so their conductance is not affected by the incoming input signal (spikes), thus their synaptic modulator output is stable. The global input to this neural pathway is represented by the spikes generated by an afferent neuron subject to high light intensity and low light intensity (Fig.3b). The pattern of the afferent neuron spikes has been previously recorded with the Arkeo System. To ensure reproducible results, the two recorded spike patterns (high light and low light intensity, Supplementary Figure 11) have been used as input using Arkeo System as a voltage supplier.

Considering Fig.3, in the case of low light intensity, when the first pump is activated, dopamine is expressed at the first synapse, so the synaptic modulator output increases (+ 0.04 mV) in turns increasing the frequency of the corresponding neuron (+ 12%). At this stage, if 5-HT is not expressed in the associated synapse, the synaptic modulator output of the last neuron does not change and no modulation of its frequency is recorded (black). When 5-HT is introduced in the artificial synapse through the microfluidic pump, a synaptic modulator output (blue, + 0.035 mV) and neuron frequency modulation (blue, +11%) is recorded.

We here contextualize the platform's performance and functions with emphasis on the presented cascaded neural pathway, establishing a direct parallel to specific biological circuits displaying similar functions and thus commenting on its applicability as a bio-interfaced system.

Building on Supplementary Discussion 3, we still consider the work by Keene et al. demonstrating the use of biohybrid synapses as devices allowing the transduction of chemical signals from PC12 cells (released dopamine) into permanent conductance modulations of the organic neuromorphic device's channel terminal. This work established a chemical to electrical signal transduction approach which has been thoroughly investigated from an electrochemical perspective by Matrone et al.[28]. In our synaptic modulator, dopamine and serotonin allow a semi-permanent conductance modulation (synaptic weight update) which is used to control the activity of a connected spiking neuron, eliciting not only frequency modulations in persistently spiking neural networks (Figs. 1 and 3) but also triggering spikes (neural activation), and so signal transmission, in a silent pathway (Fig.2). By design, the biohybrid synapse used in the synaptic modulator can be employed as an active interface with tissue and cells models. Indeed, leveraging the biocompatibility of PEDOT:PSS, cells can be directly seeded on the gate terminal of this device, with extensive reports supporting these applications in literature[38–40]. In this configuration, the presence of physiologically released electroactive neurotransmitters, such as dopamine and serotonin, allows to operate the presented neuromorphic neural pathway as an adaptive platform which does not merely record biochemical clues into electrical signals but more importantly locally transduce these relevant signals in a neuromorphic fashion.

By examining biological neural pathways involving a complex interconnection of neuromodulation processes, we establish a direct parallel between the neuromorphic platform and midbrain neurons thus validating the significance of this work.

DA neurons in the ventral tegmental area bidirectionally regulate the activity of 5-HT neurons in the dorsal raphe nucleus, a biological scenario which has been replicated through the neuromorphic platform and is illustrated in Figs. 2 and 3.[41] We investigated the biological phenomena at the base of the interaction between dopamine and serotonin neurons, and hereby highlight technological gaps which must be addressed to establish an intimate coupling between neuromorphic devices and biological pathways. First, as analyzed in detail in Supplementary Discussion 7, we reaffirm that only the amplitude of change of the spikes frequency represent the brain encoding language which allow to translate both external and internal stimuli into neural data. As such, midbrain interneurons perform computing functions through frequency modulations of +/- 30% (respect to the baseline frequency, Supplementary Fig. 30), which correspond to the same frequency changes demonstrated by our neuromorphic platform in Figs. 2 and 3. On the other hand, viable strategies to increase the operative frequency of the neuromorphic systems are investigated and suggested in Supplementary Discussion 7. Finally, focusing on the electrical to chemical signal transduction, we here investigate the

concentrations of neurotransmitters involved in biological neurons functions, to prove the neuromorphic platform applicability in bio-hybrid scenarios.

For DA and 5-HT, two kind of signalling patterns (in the midbrain and retina) have been identified as phasic and tonic, supposedly associated to the biological functions of teaching signal (direct signal processing) and motivational drive (indirect signal processing), respectively. Direct signal processing is associated to the release of neurotransmitters in the pre- to post-synaptic space (4 mm) upon pre-synaptic firing (neurons phasic activity). This mechanism represents a core computational primitive which we intended to replicate in this work. According to the suggestion that the synaptic compartment has to be distinguished from the extra-synaptic compartment, synaptic transmission can be modeled on the hypotheses that neurotransmitters release is highly localized to the synapses and that neurotransmitters uptake strongly contributes to this tight localization[42]. Under these assumptions, the concentration of neurotransmitters peaks during firing and returns to the baseline level (4-50 nM) in the ms range. While it is still difficult to estimate or directly measure the peak concentration reached during a firing event, models predict a change of the instantaneous (local) level of neurotransmitters to be in the tens of mM. In this work we used concentrations of neurotransmitters ranging from 0.025 mM to 0.1 mM (25 to 100 µM) to simulate the phasic release of dopamine and serotonin at the synaptic terminal. While lower concentrations of neurotransmitters (below 10 µM) can be still used to elicit the conductance modulation phenomena described in Supplementary Discussion 3, the range of concentrations employed in this work still correspond to a realistic biological scenario which can be replicated by bio-interfacing the platform with neuronal model cells such as PC12 (dopamine release). As such, midbrain and retina neuronal pathways depending on the interaction of afferent and interneurons mediated by the neurotransmitter dopamine and serotonin have been replicated by the design of the modular neuromorphic platform.

Supplementary Discussion 6: Power Consumption of the spiking circuit and the synaptic modulator

The spiking circuit replicating the functions of a biological neuron employs three voltage sources. Hence, the total power consumption P_{tot} can be estimated considering the single power sources contributions, i.e. the input voltage V_{IN} and the two voltages V_{DD1} and V_{DD2} that are required to operate the first and second inverter, respectively. As such, first the power for each voltage source has been calculated and then the single contributions have been summed:

$$P_i = P_i \cdot P_i \quad (5)$$

$$P_{tot} = P_{IN} + P_{VDD1} + P_{VDD2} \quad (6)$$

We evaluated the power consumption of the spiking circuit used to emulate an afferent neuron operating in both the high frequency regime (emulating the biological scenario of high light intensity) and the low frequency regime (low intensity). As shown in Supplementary Figure 24, the main power contribution is from the second inverter of the circuit (red curve).

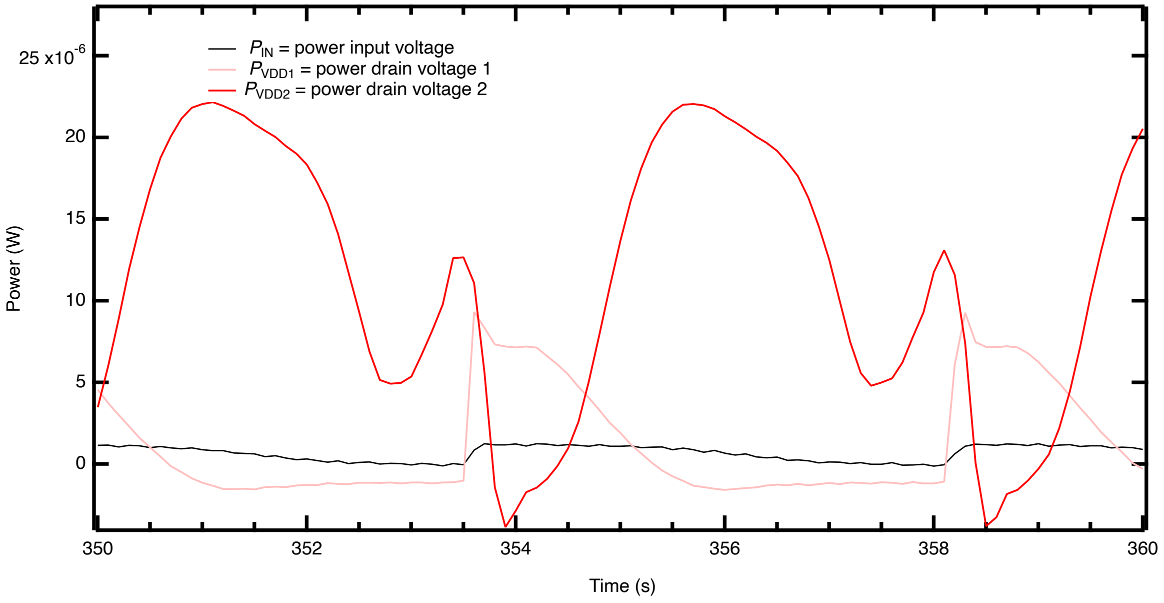

Supplementary Figure 24. Power consumption of the neuromorphic spiking circuit at high frequency. Power for each voltage source (V_{IN} black, V_{DD1} pink and V_{DD2} red) in the neuromorphic circuit high spiking regime (frequency 0.25 Hz).

The energy consumed per spike-event by the circuit was estimated by integrating the power over the time of a single spike. The energy per spike E_s consumed by the neuromorphic circuit in the high spiking-frequency regime (V_{IN} = 1.3 V, Supplementary Figure 24) is $6.2*10^{-5}$ J. On the other hand, in the low frequency regime the E_s is $1.2*10^{-4}$ J (Supplementary Figure 25). Hence, these values confirm that the neuromorphic circuit requires higher power than the one required by its biological counterpart.

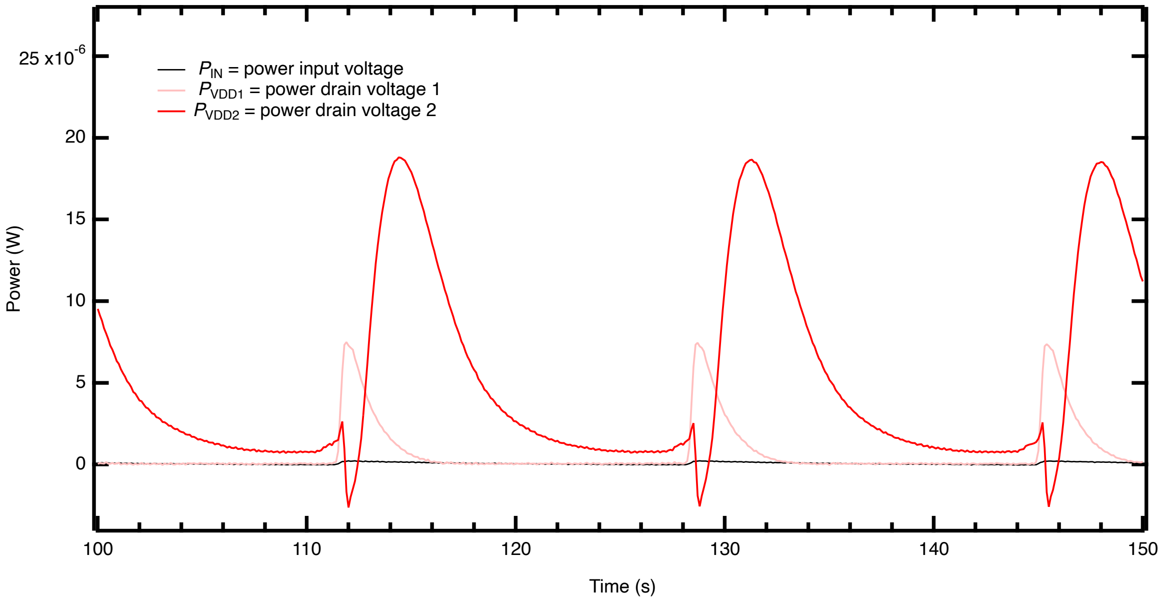

Supplementary Figure 25. Power consumption of the neuromorphic spiking circuit at low frequency Power for each voltage source (V_{IN} black, V_{DD1} pink and V_{DD2} red) in the neuromorphic circuit low spiking regime (frequency 0.10 Hz).

Indeed, in a biological circuit the energy required per spike event is significantly lower (it may vary from nJ to almost μJ)[27,43]. However, to continue the parallel with the biological circuitry, a neural signal is not only transmitted along an axon but also transferred across a synapse. As a term of comparison, 100 pJ are usually required to elicit an action potential (spike) while 10 fJ are necessary to power a synapse (synaptic transmission)[44]. Hence, we also evaluated the power consumption of a single synaptic transmission when our neuromorphic spiking circuit (neuron) is connected to an artificial synapse (corresponding synapse). The energy consumption of the spiking circuit is $1*10^{-4}$ J, evaluated as the energy required to power the artificial synapse during the course of a synaptic transmission following a spike, i.e. employing the Synaptic modulator as in Fig.1 of the manuscript, using the same strategy as above (Supplementary Figure 26). Also, in this case the energy required by the neuromorphic hardware is higher than the biological expenditure. However, we can propose a strategy to highly reduce the energy required for the synaptic transmission.

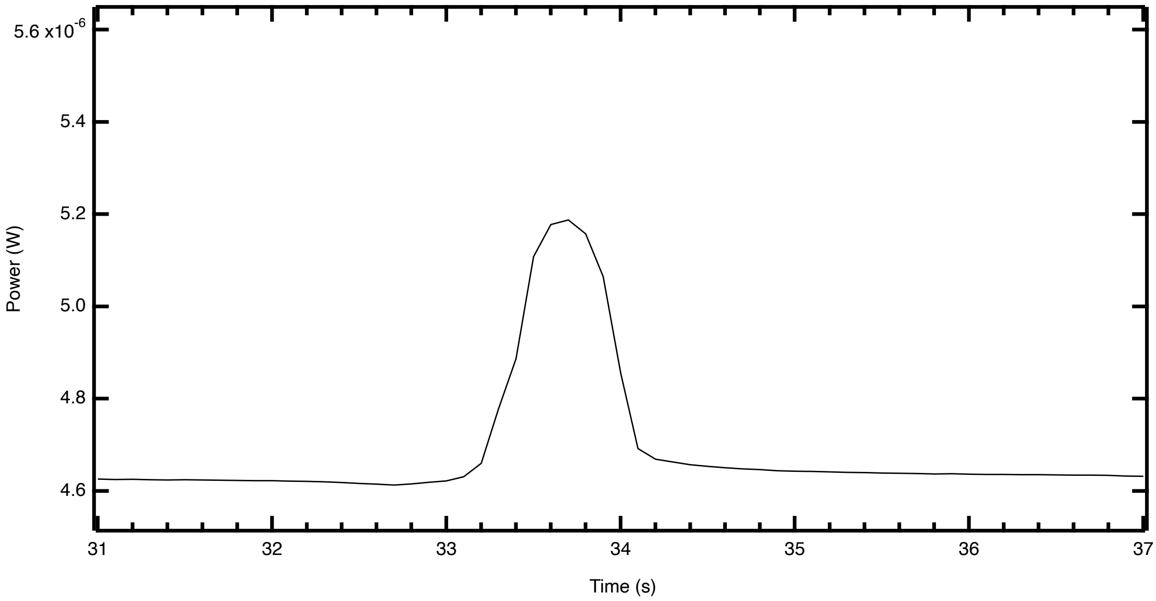

Supplementary Figure 26. Power consumption of the synaptic modulation. Power curve of the Synaptic modulator when used in connection to a neuromorphic spiking neuron.

The high energy consumption of the Synaptic modulator stems from the high V_{DD} (0.8 V) applied to its terminal which is required to keep the V_{out} of this element close to 0.2 V. Indeed this value is an operative requirement allowing the modulation of the conductance of the PEDOT:PSS-based device (bio-hybrid synapse) to induce a meaningful change of the V_{out}. Only a conductance modulation of the bio-hybrid synapse having the Synaptic modulator output close to 0.2 V can serve to trigger spikes firing on a neuron (moving the V_{out} from values below 0.2 V to values above 0.2), and to modulate the frequency of a following neuron which is already in the tonic spiking regime ($V_{out} > 0.3$), see Supplementary Figure 7. As such, to reduce power consumption the design of a bio-hybrid synapse showing a large conductance when receiving a spikes input from the afferent neuron is proposed. A possible strategy to achieve this goal is to increase the gating efficiency of this device. In particular, the gating efficiency depends on the material used to build the gate. The bio-hybrid synapse is essentially an ENODe[45] device, and its gate terminal is made of PEDOT:PSS so it cannot be replaced by the more efficient Ag/AgCl. However, alternative strategies have been already proposed, such us increasing the thickness of the gate terminal, as proved by Koutsouras et al.[46]

In this way, the V_{out} of the synaptic modulator can be lowered, by adjusting the applied V_{DD}, since the bio-hybrid synapse modulation becomes larger. Hence we developed a SPICE model to simulate the characteristic of the PEDOT:PSS based bio-hybrid synapse(Supplementary Figure 27).

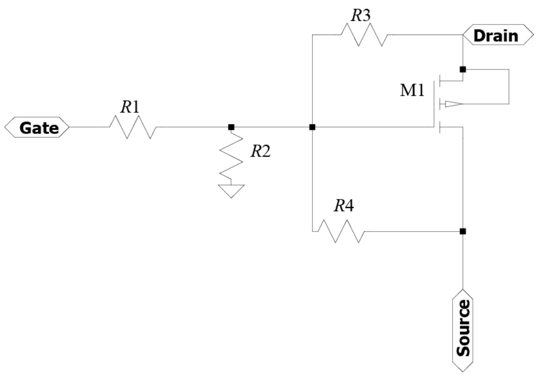

Supplementary Figure 27. The Synaptic modulator equivalent circuit in SPICE. The equivalent circuit consists of four resistors (R1 = 1 Ω, R2 = 3 kΩ, R3 = 500kΩ, R4 = 500 kΩ), and one PMOS (M1). The increased voltage drop at the device's gate is modeled via the R1-R2 voltage divider sub-circuit, where increasing R2 increases the effective voltage drop at the gate thereby mimicking the effect of varying the PEDOT thickness on the gate.

It consists of four resistors (R1= 1 Ω, R2 = 3 kΩ, R3 = 500 kΩ, R4 = 500 kΩ), and one PMOS (M1). The increased voltage drop at the device's gate is modeled via the R1-R2 voltage divider sub-circuit, where increasing R2 increases the effective voltage drop at the gate thereby mimicking the effect of varying the PEDOT thickness on the gate.

The value of R2 that allows to simulate the characteristics of the real device used in the manuscript is 6 kΩ, our baseline device. Hence, we decreased R2 resistance to simulate the effects of an increased gating efficiency, i.e minimizing the voltage drop at the gate/electrolyte interface while maximizing the drop at the channel interface, a phenomenon that can be induced by increasing the gate terminal thickness[46].

Supplementary Figure 28 shows that the change induced by the input of an afferent neuron on the V_{out} is amplified by increasing the gate efficiency (decreasing the value of R2, black arrow). This allows to adjust, as from the plot, the V_{DD} of the voltage divider and consume less power-per-spike.

We then evaluated the power consumption for each of the different values of R5 tested and derived a general trend for the reduction of this metric. Indeed, the lowest simulated value of R2 (0.1 kΩ) allows to reduce the power consumption of the synaptic modulator and so of the artificial synaptic transmission from $1*10^{-4}$ J (related to R2 = 6 kΩ) to $6.93*10^{-7}$ (Supplementary Figure 29). We believe this strategy, combined with the classic approach of device-scaling which permit to decrease the voltage divider V_{DD}, should allow the next generation of bio-hybrid synapses to approach the energy consumption window of their biological counterpart (10 fJ). The device-scaling approach, given the starting size of the bio-hybrid synapse system, can be pursued without the disadvantages of noise increase and reliability reduction which are usually relevant in case of size reduction below the micrometre scale. On the other hand, a foreseeable challenge is represented by the development of a scaled version of the fluidic module to locally control the concentration of neurotransmitters. However, research on microfluidic modules is starting to develop innovative approaches for

integration of these systems with devices comprising a lateral footprint close 1mm, with advanced optical, mechanical, electrical chemical, and bio-interfacial properties[47].

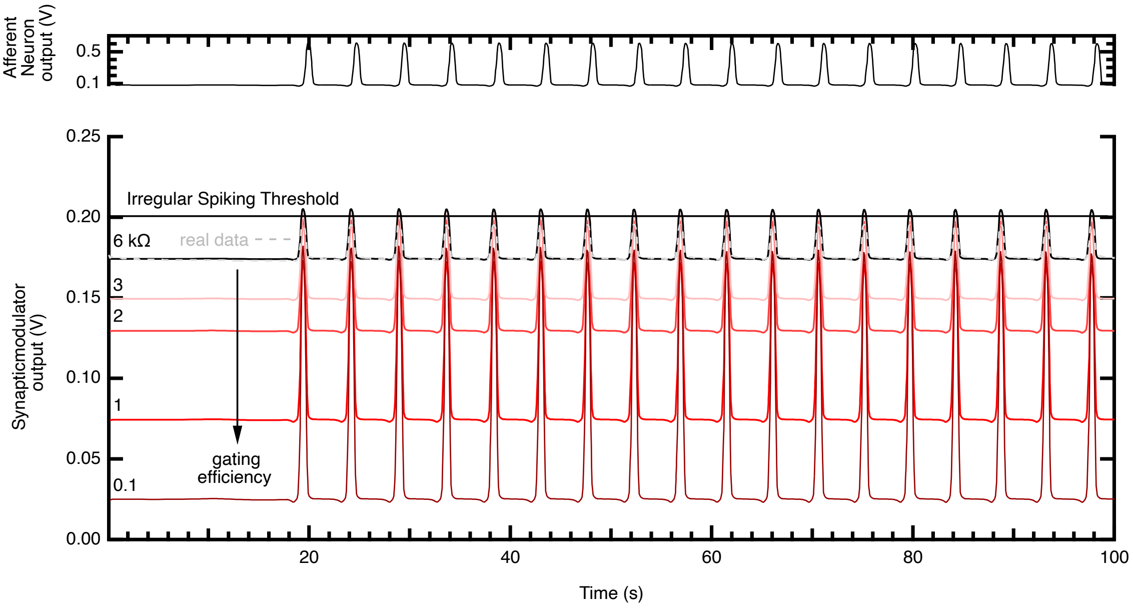

Supplementary Figure 28. Synaptic modulator outputs from simulations. Synaptic modulator outputs from the simulation of biohybrid synapses with increasing gating efficiency (from 6 kΩ to 0.1 kΩ). For each different gating efficiency, the V_{DD} of the Synaptic modulator is adjusted to allow the V_{OUT} to reach the value of 0.2 V (irregular spiking threshold) under the activity of the afferent neuron.

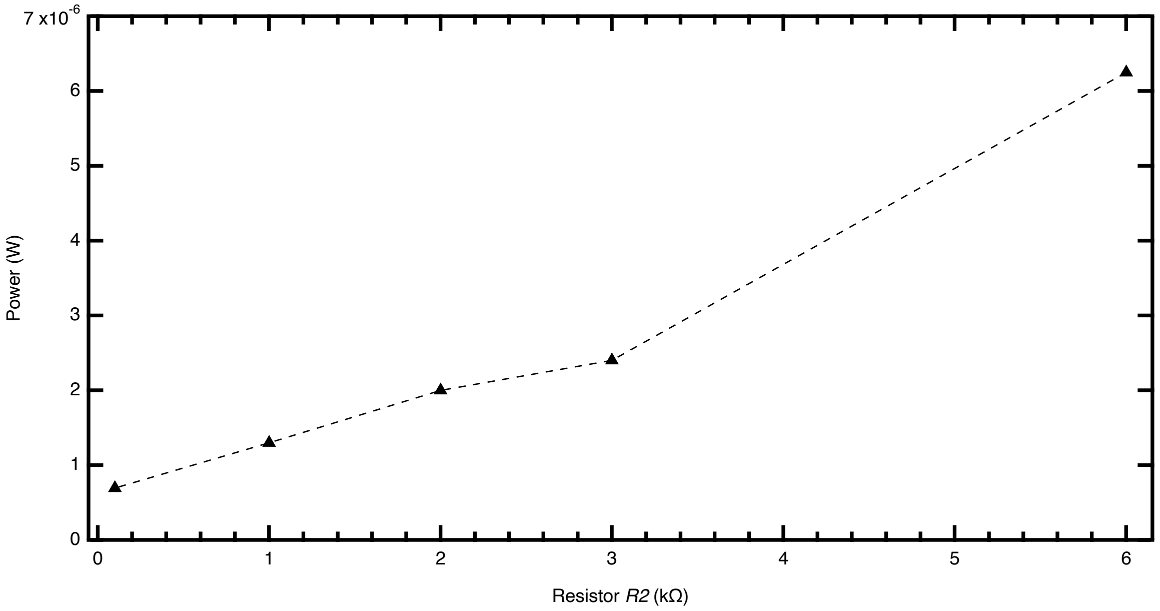

Supplementary Figure 29. The role of the resistance R2. Relation between the resistance of R2 and the energy consumption of the Synaptic modulator operated in the voltage window shown in Supplementary Figure 28.

Supplementary Discussion 7: Increasing the frequency of the spiking circuit.

Biological neurons, especially in the human body, display a plethora of spiking responses to the external stimuli, i.e. different neurons produce multiple spikes in response to an input pulse due to specific design constraints. As such, to emulate a biologically plausible scenarios, an artificial neuron must implement diversified responses to ensure reliable communication and synchronization to the other neurons of the network. Due to the high specialization of neural cells, the frequency range of neurons ranges from sub-Hz to almost 500 Hz[27,48,49]. Also, neurons are highly specialized to exhibit a specific frequency modulations that are crucial for neural synchronization and so for the computing functions of the brain[49].

For example, when dopamine is released in the striatum or in the ventral tegmental area it can activate D1-like receptor on the neurons in the pathway. These dopaminergic neurons usually fire in a low range of frequencies (0.5 -10 Hz)[50]. In general, dopamine's effect on the spike frequency of neurons can be described in terms of relative changes (frequency increase/decrease by a certain percentage compared to the baseline frequency). However, the specific rate of variations and the time over which these occur can vary widely depending on the experiments performed (in vivo, implants, etc etc) and the physiological environment. In the context of this manuscript, we focus on replicating human retinal pathway. Although the dopaminergic amacrine cells response to light stimuli involves complicated processes, generally light leads to an increase of the dopamine levels in this pathway which can be associated with an increase of firing activity. Indeed, dopamine (DA) is the key neuromodulator that in the retina adjusts the circuitry for visual processing in dim and bright light conditions. As matter of fact, along these neural pathways, light triggers the neural activity which is further regulated by the concentration of dopamine. Whilst neurons in these cases display spontaneous spike activity ranging from silent to rhythmic to periodic burst discharge, recording from amacrine cells proved their light sensitivity generating responses that varied with intensity. As anticipated, the specific frequency variations reported in literature are sparse and depends on the measurement techniques and the specific experiment performed. However, in such neural ensembles which operate at the frequencies below 10 Hz, deviations as low as 0.02 Hz in spike frequency between groups successfully alter the phase synchrony allowing dynamic processes at the base of computation[49,51,52].
Indeed, in this study we have shown frequency modulations up to ≈ 30% (Fig.3) of the baseline spike frequency which are close to ones reported in Supplementary Figure 30.

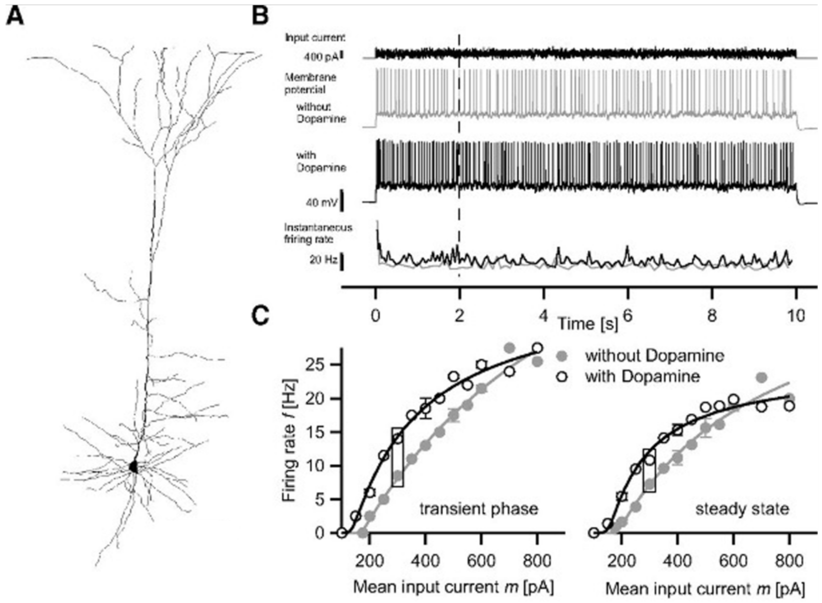

Supplementary Figure 30. Dopamine role in the prefrontal cortex neurons. Dopamine differentially affects layer 5 pyramidal neurons in the prefrontal cortex (PFC). A: reconstruction of a layer 5 PFC pyramidal neuron. The scale bar is 100 µm. B) example of the noisy current stimulus with a mean of 250 pA and corresponding voltage traces without and with dopamine application. The corresponding instantaneous firing rate is also reported. C) example of the influence of dopamine on the response curve of a layer 5 PFC pyramidal neuron. Curves are given for the transient phase at the beginning of the stimulation (transient phase) and for the steady-state response (steady state).

The neuromorphic spiking circuit has a maximum frequency of 0.25 Hz, and all the frequency modulations here reported are in the range of 0.08 to 0.25 Hz, corresponding to a + 1-30% increase (Supplementary Table 2). Only few neurons in the human body spike below 1 Hz and can be deemed relevant to replicate a retinal neural pathway. However, our work focuses on the modulation of the frequency patterns (rather than on the spiking frequency optimization) as an essential bio-inspired functions to impart to the system a computational primitive for processing capabilities with a modular and reconfigurable architecture for the design adaptive network.
Indeed, the spike frequency modulation enables to emulate the mechanisms of biological signal transmission and computation: a promise for the next generation of neuromorphic processing units and the application of pre-processing algorithms. On the other hand, since a baseline frequency below 1Hz might still constitute a limit to future tissue coupling, we analyze here the neuromorphic spiking circuit dynamic providing an approach to increase its operative frequency.

The frequency of the neuromorphic spiking circuit is limited by the response time of the OECTs of the inverters and the switch. Also, the charging time of the internal capacitance of these elements as well as the capacitor must be considered. These are in general the dominant parameters affecting the dynamic of the circuit.
We developed a SPICE model to simulate the behavior of the system under the previous experimental conditions and trace the route to a meaningful and achievable increase of the spike frequency.

The typical rise time for the ambipolar OECTs used in the inverters of the circuit is around 120 ms, while the P3O-based switch exhibits a faster response of approximately 55 ms. We extracted the response time for the two materials from the curves reported in Supplementary Figure 31, using an exponential function to perform the fit (black). In this condition, the total delay would be around 50 ms (summing the delays of the inverter pair and the switch) which translates into a max frequency of 3.4 Hz. The maximum frequency (with V_{IN} = 1.3 V) recorded from this circuit is 0.25 Hz, which is clearly not in line with the expected delays and devices responses. To understand the dynamic of the circuit, we must consider the contribution of the OECTs internal capacitance whose charging time dominates the spike delays. We note that the response time and capacitance of the PEDOT:PSS-based capacitor can be excluded from this estimate, since the capacitance of the other elements (as we prove in this section with our model) is dominant.

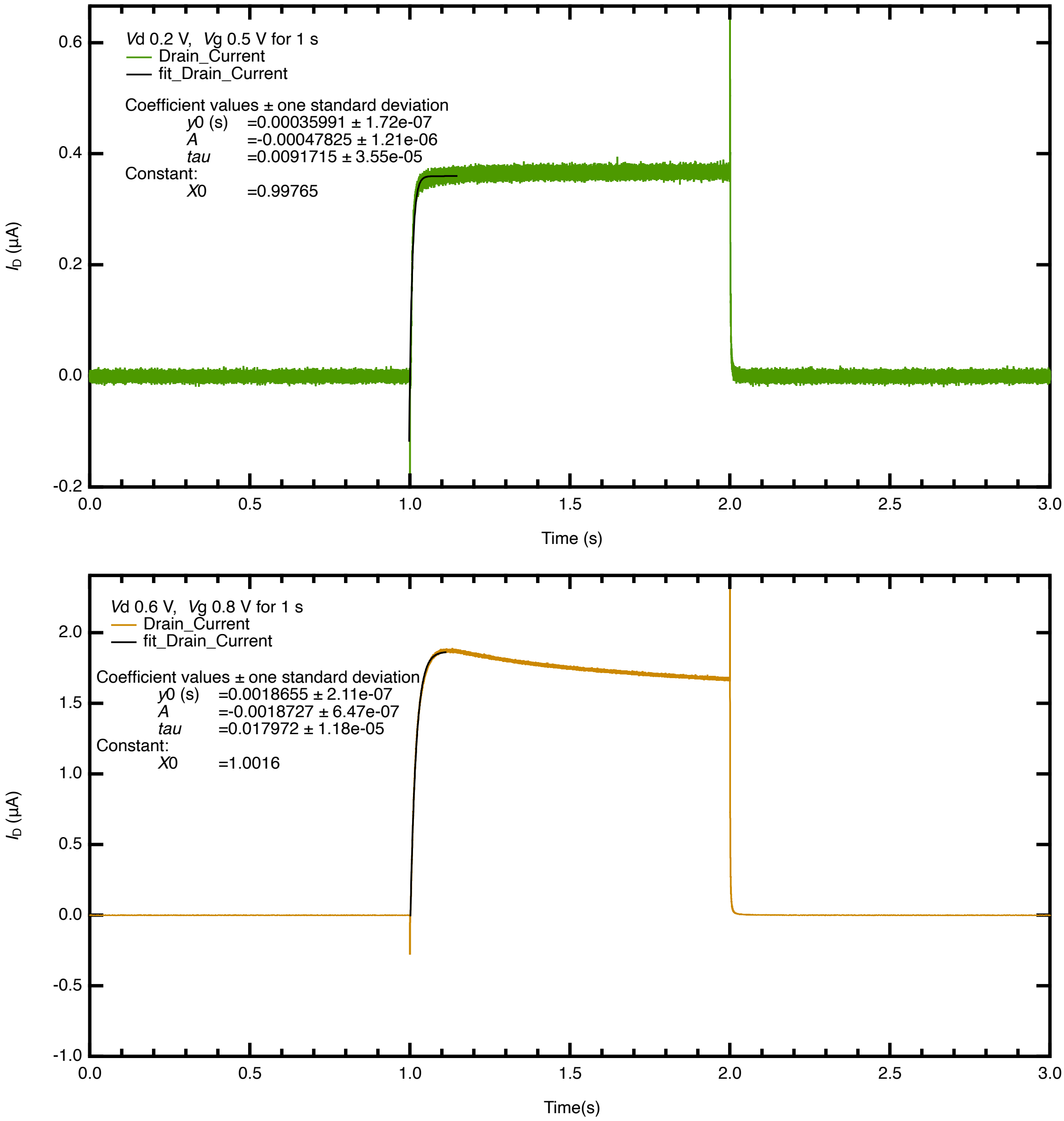

Supplementary Figure 31. Response time of key circuit component. Response time experiments for the OECT comprising the inverters (green curve) and the switch OECT (brown curve). The curves are fitted with an exponential function (in black).

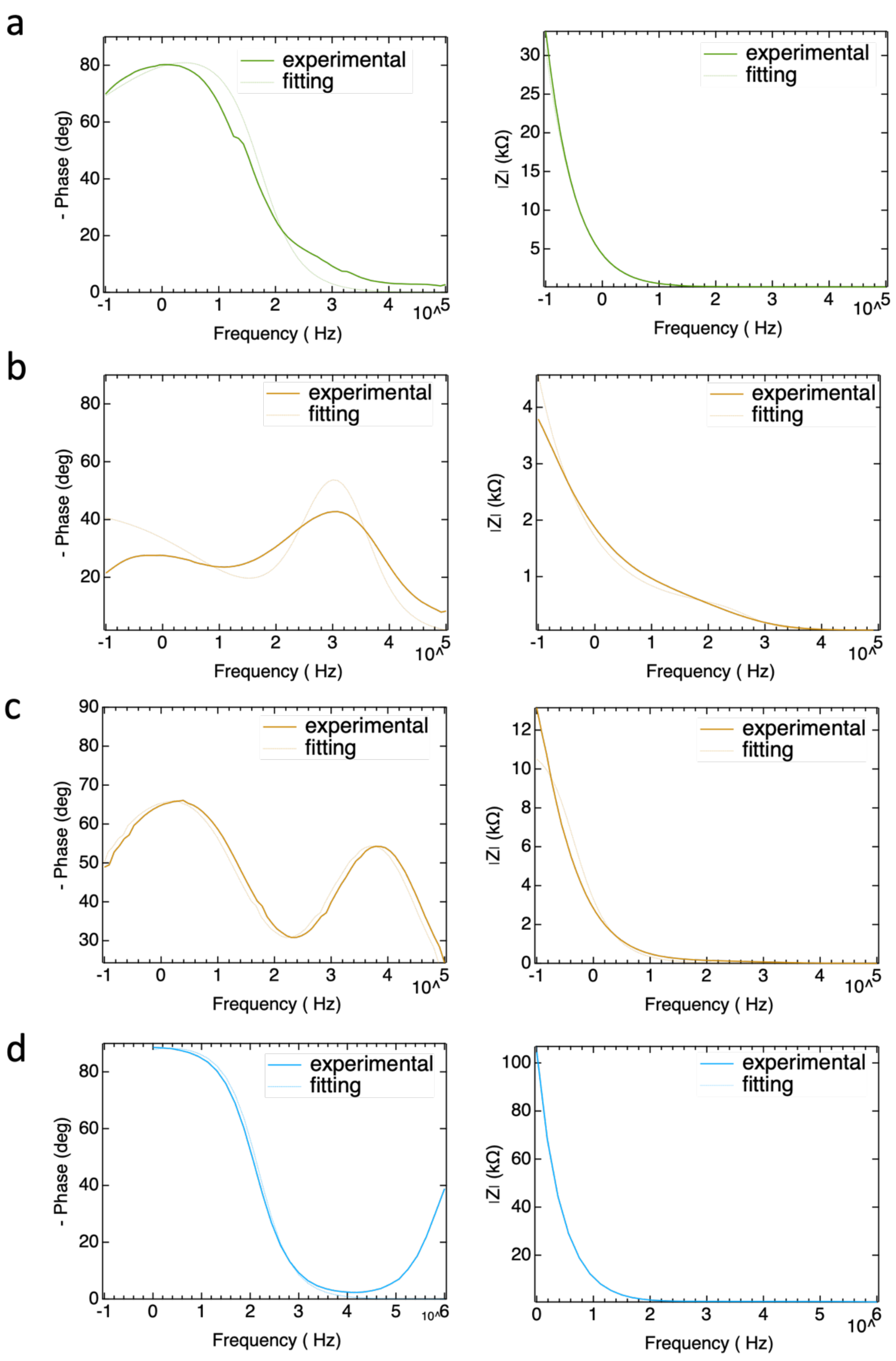

Supplementary Figure 32. EIS measurement on different circuit elements. Bode plot and phase angle of a) p(C4-T2-C0-EG) with d = 150nm in PBS at the working potential of V = 0.4 V, b) P3O with d =150 nm in PBS at the working potential of V = 0.6V, c) P-3O in a vertical OECT architecture with .d =150 nm in PBS at the working potential of *V* = -0.6 V, d) PEDOT:PSS with d = 150 nm in PBS at the working potential of *V* = - 0.2 V a,b,d) the device dimension are the same used for the spiking circuit (see Methods) and Supplementary Discussion 1.

To achieve a biologically plausible spiking regime with the architecture of our spiking circuit, the capacitances and so the response times of the above-mentioned elements must be reduced. We calculated the capacitance of the OECTs comprising the three different elements of the circuit, using impedance spectroscopy (Supplementary Figure 32, a,b,d), since these values rule the response time of the devices and so of the circuit. For the circuit used in this study, the capacitances of the different elements are reported in Supplementary Table 9. Hence, we modelled in SPICE the inverter and switch OECTs using the equivalent circuits reported in Supplementary Figure 33 and Supplementary Figure 34, respectively.

The equivalent circuit for the inverter OECT consists of four resistors (R1 = 10 kΩ, R2 = 1kΩ, R3 = 500 kΩ, R4 = 800 kΩ), one capacitor (C1 = 7 μF), one diode (D1), one PMOS (M1), and one NMOS (M2). The ambipolarity is achieved via an in-parallel connection of a PMOS and NMOS transistor tuned to appropriate thresholds and transconductances. Furthermore, device hysteresis is tuned via adjusting the R1, R2, and *C*1 values, while gate leakage is tuned via R3 and R4.
The equivalent circuit for the switch OECT consists of four resistors (R1= 10 kΩ, R2 = 1 kΩ, R3 = 500 kΩ, R4 = 500 kΩ), one capacitor (C1 = 10 μF), one diode (D1), and one NMOS (M2).

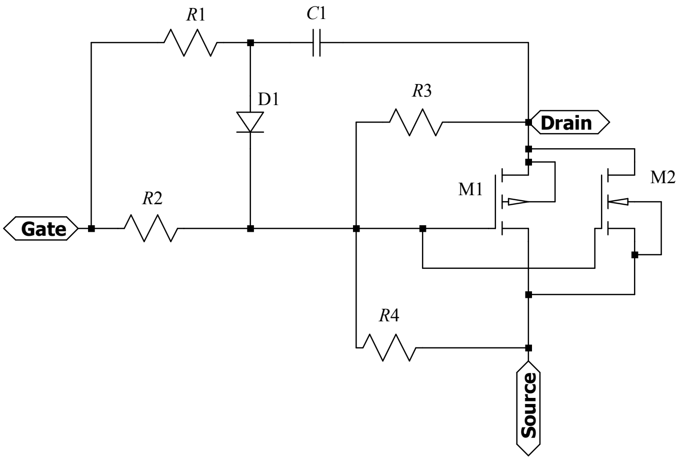

Supplementary Figure 33. Inverter OECT equivalent circuit in SPICE. The schematic depicts the equivalent circuit consisting of the resistors R1, R2, R3, R4, the diode D1, the capacitor C1 the transistors M1, M2.

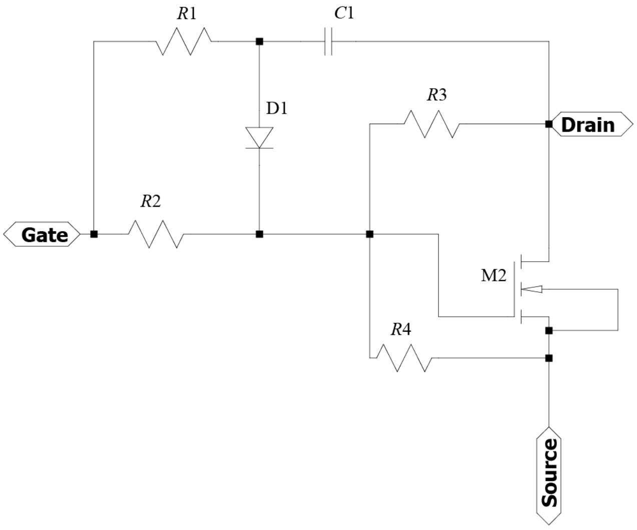

Supplementary Figure 34 Switch OECT equivalent circuit in SPICE. The schematic depicts the equivalent circuit consisting of the resistors R1, R2, R3, R4, the diode D1, the capacitor C1 the transistor M2.

Supplementary Table 9. Simulations of different capacitance values. Values for the capacitance of the different elements of the studied neuromorphic circuit, as extracted from impedance spectroscopy measurements of Supplementary Fig.33. Also, the capacitances of the 2% reduced model circuit used to simulate a fast-spiking circuit are reported.

	Capacitance (µF) experimental circuit	Capacitance (µF) 2% reduced model circuit
Inverter OECTs p(C4-T2-C0-EG)	7	0.14
Switch OECT P3O	10	0.2
Capacitor PEDOT:PSS	1	0.02

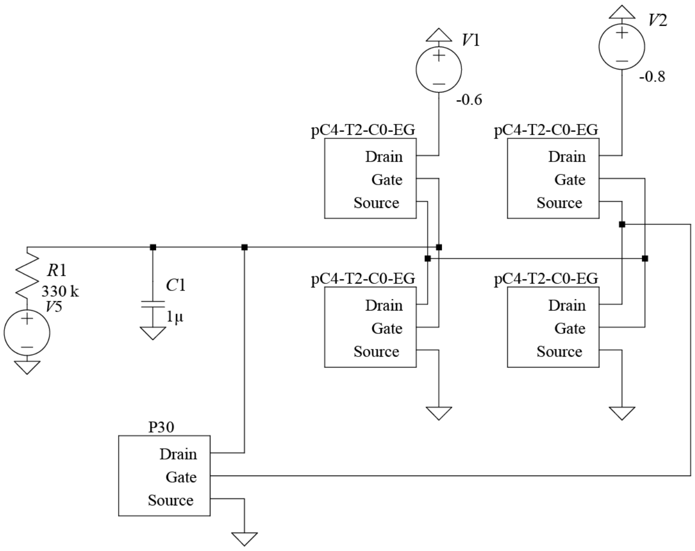

Supplementary Figure 35. Spiking circuit equivalent model in SPICE. The schematics depict the equivalent circuit consisting of the resistor R1, the capacitor C1 the switch device (P3O), the two invert pairs (p(C_4-T2-C_0-EG)) connected to the drain voltages V1 and V2.

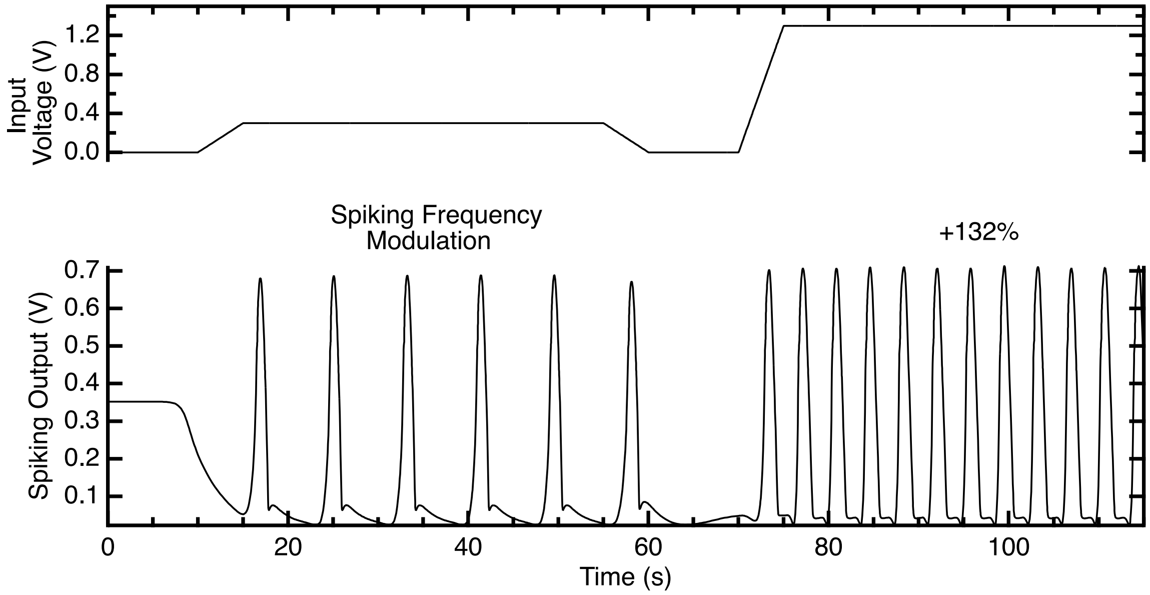

Supplementary Figure 36. Spikes from simulations. The spikes generated by the SPICE modelled circuit responding to the input conditions (high and low light intensity) used in Supplementary Figure 11.

Indeed, using the capacitance values in Supplementary Table 9 we build a SPICE model for the spiking circuit (Supplementary Figure 35), and we were able to closely simulate the behavior of experimental spiking circuit, obtaining the same spikes feature, baseline spike frequency and modulation (Supplementary Figure 36). To increase the spike frequency of this SPICE modelled circuit we

decreased the capacitance of the OECTs comprising the inverters OECTs, since these constitute the dominant factor.

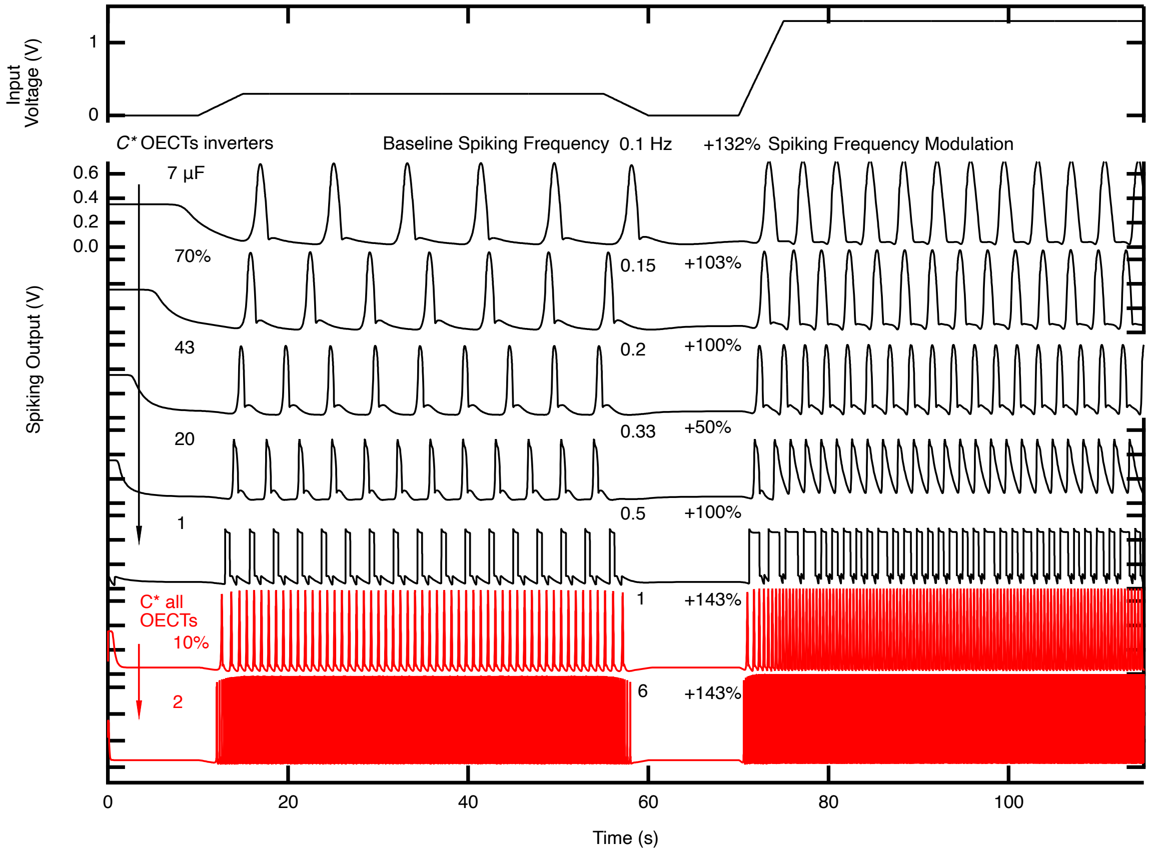

Supplementary Figure 37. Spike frequency dependence on capacitance values. The spikes generated by the SPICE modelled circuit responding to the input conditions (high and low light intensity) used in Supplementary Figure 11. The capacitance of the OECTs comprising the inverter pair is reduced as a strategy to increase the circuit spike frequency from 7 to 1.5 µF (black arrow and black curves). To achieve biologically relevant frequencies, the capacitances of all the elements have been reduced to 10% and 2% of their initial values (red arrow and red curves). In the middle of the graph the baseline frequency (Vin = 0.3V) of each iteration of the circuit and the corresponding modulation achieved at max frequency (Vin = 1.3V) are reported.

Supplementary Table 10. Simulations capacitance values. Values for the capacitance of p(C4-T2-C0-EG)-based OECT, used to run the SPICE simulation showing increasing spike frequency (black arrow, black curves Supplementary Figure 37)

Capacitance p(C4-T2-C0-EG) [µF]	Frequency [Hz] @ Vin = 0.3V	Frequency [Hz] @ Vin = 1.3V	Frequency change %
7	0.11	0.26	132,5
5 (70%)	0.15	0.31	103,1
3 (43%)	0.2	0.4	100

1.5 (20%)	0.33	0.5	50
0.01 (1%)	0.5	1	100

Supplementary Table 11. Simulations capacitance values for higher spike frequency. Values for the capacitance of the circuital elements used to run the SPICE simulation showing increasing spike frequency (red arrow, red curves Supplementary Figure 37)

Capacitance circuit elements	Frequency [Hz] @ *V*in = 0.3V	Frequency [Hz] @ *V*in = 1.3V	Frequency change %
10%	1,17	2,85	142,85
2%	5,88	14,2	142,85

Supplementary Figure 37 (black arrow, black curves) and Supplementary Supplementary Table **10** shows how decreasing the value of capacitance of the inverter OECTs from 7 to 1.5 µF allow to increase the baseline frequency (*V*in = 0.3V) from 0.1Hz to 0.5Hz. However, we observe that with this approach the biological spike frequency window (1-10 Hz) could not be approached. To further increase the frequency, we used again the same equivalent circuit and decreased the different elements capacitance values to 10% and 2% of their initial values (Supplementary Table 7 and Supplementary Supplementary Table **10**).

In this case, the baseline frequencies increase to 1 and 5 Hz for the 10% and 2% capacitance circuits, respectively (Supplementary Figure 37, red arrow and red curves). Moreover, increasing the *V*in to 1.3V the modulations of frequency match the experimental circuit one and so the biological plausible scenario.

Hence, we explored strategies to experimentally achieve such low values of capacitance working on the architecture of the single devices. In case of the PEDOT:PSS capacitor we fabricated planar devices with reduce electrode area proving how by device-scaling the capacitance of this element can be easily tuned from 8.7 µF to 3.62 nF (Supplementary Table 12).

Supplementary Table 12. Values for the capacitance of PEDOT: PSS devices. Device capacitance values with decreasing area, as extracted from impedance spectroscopy measurements. The extracted capacitance values are averaged over three devices.

Capacitor PEDOT:PSS	Area (m)	Capacitance (uF) from EIS
250 × 500 µm device (integrated neuron)	$1.25\ 10^{-7}$	1 ± 0.1

50 × 50 μm device	1.5 10^{-9}	0.4 ± 0.03
20 × 20 μm device	1.5 10^{-10}	0.01 ± 0.004

In order to decrease the capacitance of the OECTs, due to the high resistive nature of both the materials used in this study (p(C4-T2-C0-EG) and P3O) requiring an interdigitated design, we could not achieve the low capacitance values reported in Supplementary Table 11 by conventional device-scaling. However, as a model system we used P3O to fabricate vertical OECTs and check their capacitance[53]. With this architecture, the thickness of the ambipolar polymer film (150 nm) represents the channel length of the transistor, while the width can be controlled by the bottom electrode area (in this case 50 × 50 μm).

Performing impedance spectroscopy on a series of three devices (Supplementary Figure 32), we extracted values of capacitance in the range 0.05 to 0.11 μF, matching the target values highlighted by the circuit model. With the same vertical architecture, we can reduce the capacitance of the inverter OECTs to 0.07 μF. As such, we hereby provide a suitable strategy to increase the frequency of the neuromorphic spiking circuit, keeping the same circuit design and working on the dimension and architecture of the single elements.

The spiking circuit frequency can be increased above the previously mentioned values, achieving the 50-100 Hz window (which can be interesting for additional physiological functions emulation), by further reducing the capacitances of all the circuital elements. However, in this case the required capacitance values could not be achieved with the combination of materials employed in this study by downscaling the single devices or adopting a vertical architecture (as from above). Nonetheless, in Supplementary Figure 38 we report the frequency increase with the reduction of the capacitance (in percentage respect to the real circuit capacitances reported in Supplementary Table 9) in case of a reduction below 10%. In order to achieve a frequency value of 100 Hz, the capacitance of all the elements should be reduced to 0.33%, corresponding to 0.03 nF (P3O), 0.023 nF ((p(C4-T2-C0-EG)) and 0.003 nF (PEDOT:PSS). With the use of different OECTs material showing an intrinsic reduced capacitance, in combination with the vertical OECT architecture, in future high spiking frequencies circuits can be fabricated.

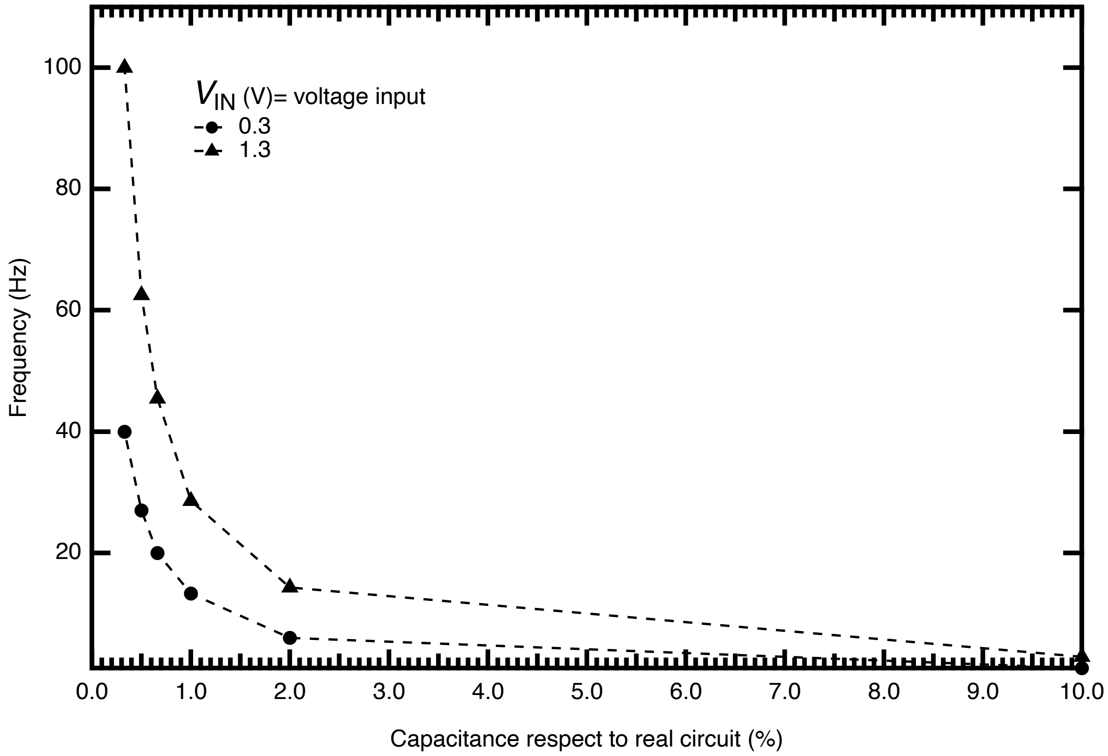

Supplementary Figure 38. Spike frequency VS capacitance plot. The capacitances used for the simulated circuits are expressed as percentages respect to the values of capacitance extracted from the real circuit and reported in Supplementary The schematic depicts the equivalent circuit consisting of the resistors R1, R2, R3, R4, the diode D1, the capacitor C1 the transistor M2.

We lastly checked the energy consumption of this circuit, from the simulation voltage and current traces (Supplementary Figure 39). The experimental circuit used in the study, as from Supplementary discussion 7, has an energy consumption of $6.2*10^{-5}$ J per spike. The simulated fast spiking circuit achieve and energy consumption of $8.9*10^{-7}$ J, a remarkable decrease of almost two orders of magnitude. We note that by downscaling the OECTs of the circuit using the vertical architecture we also achieve a reduction of the needed operational voltages V_{DD1} and V_{DD2} which can further decrease the energy consumption.

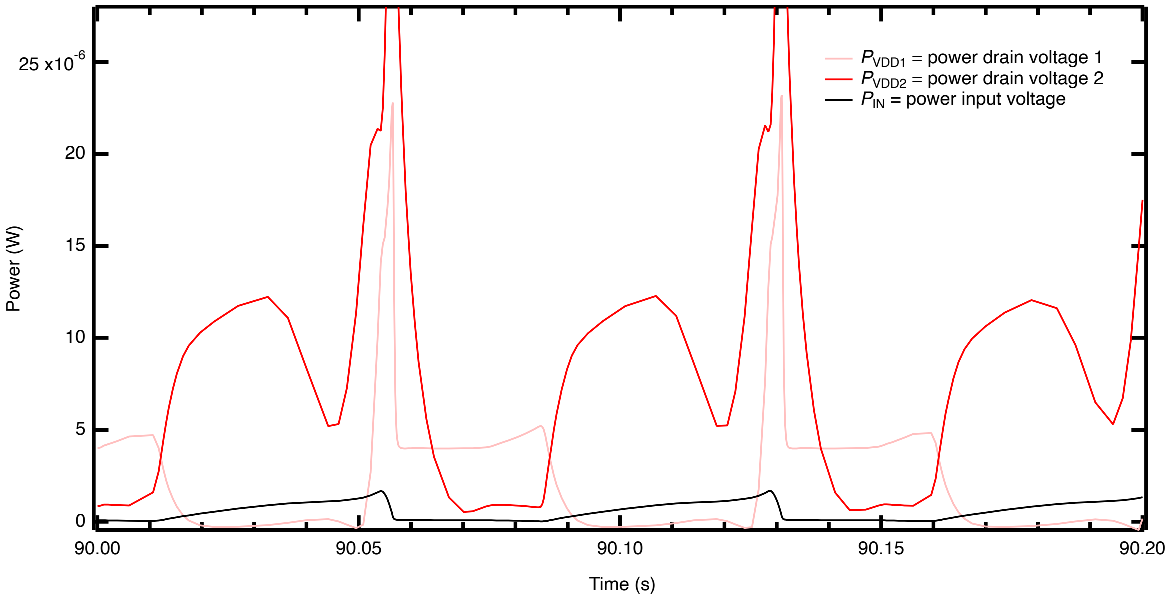

Supplementary Figure 39. Power consumption for the best simulated circuit. Power for each voltage source (V_{IN} black, V_{DD1} pink and V_{DD2} red) in the simulated neuromorphic circuit (capacitance 1%) in the high spiking regime (frequency 14.2 Hz).

Supplementary Discussion 8: Performance comparison of artificial neurons based on different technologies.

We report in Supplementary Table 1 (Supplementary Discussion 1) useful parameters to describe the performance of artificial neurons based on both silicon and organic materials. Examining the most recently published artificial neuron systems, we focus our analysis on the parameters we deem relevant to demonstrate the relevance of these platforms in replicating biological neurons. We highlight that Si-based technologies still represent the state-of-the-art systems considering power consumption per spike and spike voltage amplitude. Due to the programming flexibility, these devices display the highest number of biologically relevant features is reported. However, they only organic based systems show a certain degree of tunability in ionic environment, combining sensing and actuation. Evidently, this is a critical characteristic that allows for future bio-interfacing, even though the operational frequency is still lower than biological neurons. Among organic systems, Hodgkin-Huxley (HH) models are emerging due to the possibility to closely emulate the biological spike features (Na+ and K+ channels function) and to operative frequency matching the biological window. However, these latter and most organic neuromorphic systems are based on single devices or simple circuits which exhibit limited adaptability to both external and internal biologically relevant cues, and are restricted to emulate only specific biological functions of an individual neuron or synapse (no connectivity to additional neurons). Especially for HH model systems, connectivity between more than one neuron and artificial synapses

is complicated by an intrinsic mismatch of the output-input signals of these devices. Indeed, HH systems operate receiving as input a current which must be contained in a narrow window to allow spiking activity and avoid saturation. As such, artificial synapses which normally produce a voltage output might not represent the ideal input to HH spiking neurons. Hence, in this work we employed the leaky-integrate-and-fire LIF architecture which allowed us to build a modular neuromorphic system where the connections between synapses and neurons can be re-arranged to replicate specific neural pathways.

Supplementary References

1. Serb, A. *et al.* Memristive synapses connect brain and silicon spiking neurons. *Scientific Reports* **10**, 1–7 (2020).
2. Gumyusenge, A., Melianas, A., Keene, S. T. & Salleo, A. Materials Strategies for Organic Neuromorphic Devices. *Annu. Rev. Mater. Res.* **51**, 47–71 (2021).
3. Paulsen, B. D., Tybrandt, K., Stavrinidou, E. & Rivnay, J. Organic mixed ionic–electronic conductors. *Nature Materials* **19**, 13–26 (2020).
4. Simon, D. T., Gabrielsson, E. O., Tybrandt, K. & Berggren, M. Organic Bioelectronics: Bridging the Signaling Gap between Biology and Technology. *Chem. Rev.* **116**, 13009–13041 (2016).
5. Owens, R. M. & Malliaras, G. G. Organic Electronics at the Interface with Biology. *MRS Bulletin* **35**, 449–456 (2010).
6. Bettucci, O., Matrone, G. M. & Santoro, F. Conductive Polymer-Based Bioelectronic Platforms toward Sustainable and Biointegrated Devices: A Journey from Skin to Brain across Human Body Interfaces. *Adv. Mater. Technol.* 2100293 (2021) doi:10.1002/admt.202100293.
7. Akbari, M., Hussein, S. M., Chou, T.-I. & Tang, K.-T. A 0.3-V Conductance-Based Silicon Neuron in 0.18 μm CMOS Process. *IEEE Trans. Circuits Syst. II* **68**, 3209–3213 (2021).
8. Livi, P. & Indiveri, G. A current-mode conductance-based silicon neuron for address-event neuromorphic systems. in *2009 IEEE International Symposium on Circuits and Systems* 2898–2901 (IEEE, Taipei, Taiwan, 2009). doi:10.1109/ISCAS.2009.5118408.
9. Wijekoon, J. H. B. & Dudek, P. Compact silicon neuron circuit with spiking and bursting behaviour. *Neural Networks* **21**, 524–534 (2008).

10. Rubino, A., Payvand, M. & Indiveri, G. Ultra-Low Power Silicon Neuron Circuit for Extreme-Edge Neuromorphic Intelligence.

11. Vuppunuthala, S. & Pasupureddi, V. S. 3.6-pJ/Spike, 30-Hz Silicon Neuron Circuit in 0.5-V, 65 nm CMOS for Spiking Neural Networks. *IEEE Trans. Circuits Syst. II* 1–1 (2023) doi:10.1109/TCSII.2023.3324584.

12. Pickett, M. D. A scalable neuristor built with Mott memristors. *NATURE MATERIALS* **12**, (2013).

13. Yi, W. *et al.* Biological plausibility and stochasticity in scalable VO2 active memristor neurons. *Nat Commun* **9**, 4661 (2018).

14. Li, X. *et al.* A Memristors-Based Dendritic Neuron for High-Efficiency Spatial-Temporal Information Processing. *Adv. Mater.* (2023).

15. Amin Fida, A., Khanday, F. A. & Mittal, S. An active memristor based rate-coded spiking neural network. *Neurocomputing* **533**, 61–71 (2023).

16. Zhao, J. *et al.* Memristors based on $NdNiO_3$ nanocrystals film as sensory neurons for neuromorphic computing. *Mater. Horiz.* **10**, 4521–4531 (2023).

17. Thakar, K., Rajendran, B. & Lodha, S. Ultra-low power neuromorphic obstacle detection using a two-dimensional materials-based subthreshold transistor. *npj 2D Mater Appl* **7**, 68 (2023).

18. Beck, M. E. *et al.* Spiking neurons from tunable Gaussian heterojunction transistors. *Nat Commun* **11**, 1565 (2020).

19. Yan, X. *et al.* Reconfigurable mixed-kernel heterojunction transistors for personalized support vector machine classification. *Nat Electron* (2023) doi:10.1038/s41928-023-01042-7.

20. Mirshojaeian Hosseini, M. J. *et al.* Organic electronics Axon-Hillock neuromorphic circuit: towards biologically compatible, and physically flexible, integrate-and-fire spiking neural networks. *J. Phys. D: Appl. Phys.* **54**, 104004 (2021).

21. Mirshojaeian Hosseini, M. J. *et al.* An organic synaptic circuit: toward flexible and biocompatible organic neuromorphic processing. *Neuromorph. Comput. Eng.* **2**, 034009 (2022).

22. Tischler, V. An integrate-and-fire neuron circuit made from printed organic field-effect transistors. *Organic Electronics* (2023).

23. Harikesh, P. C. *et al.* Organic electrochemical neurons and synapses with ion mediated spiking. *Nat Commun* **13**, 901 (2022).
24. Wu, H.-Y. *et al.* Stable organic electrochemical neurons based on p-type and n-type ladder polymers. *Mater. Horiz.* **10**, 4213–4223 (2023).
25. Zhang, Y. *et al.* Adaptive Biosensing and Neuromorphic Classification Based on an Ambipolar Organic Mixed Ionic–Electronic Conductor. *Advanced Materials* **n/a**, 2200393.
26. Ohayon, D. *et al.* Influence of Side Chains on the n-Type Organic Electrochemical Transistor Performance. *ACS Appl. Mater. Interfaces* **13**, 4253–4266 (2021).
27. Kandel, E. R., Schwartz, J. H., Jessell, T. M., Siegelbaum, S. A. & Hudspeth, A. J. *Principles of Neural Science*. vol. 4 (McGraw-hill New York, 2000).
28. Matrone, G. M. *et al.* Electrical and Optical Modulation of a PEDOT:PSS-Based Electrochemical Transistor for Multiple Neurotransmitter-Mediated Artificial Synapses. *Adv Materials Technologies* 2201911 (2023) doi:10.1002/admt.202201911.
29. Rivnay, J. *et al.* Organic electrochemical transistors. *Nature Reviews Materials* **3**, (2018).
30. Keene, S. T. A biohybrid synapse with neurotransmitter-mediated plasticity. *Nature Materials* **19**, 16 (2020).
31. Shahrokhian, S. & Bozorgzadeh, S. Electrochemical oxidation of dopamine in the presence of sulfhydryl compounds: Application to the square-wave voltammetric detection of penicillamine and cysteine. *Electrochimica Acta* **51**, 4271–4276 (2006).
32. Huether, G., Fettkötter, I., Keilhoff, G. & Wolf, G. Serotonin Acts as a Radical Scavenger and Is Oxidized to a Dimer During the Respiratory Burst of Activated Microglia. *Journal of Neurochemistry* **69**, 2096–2101 (2002).
33. Sarada, B. V., Rao, T. N., Tryk, D. A. & Fujishima, A. Electrochemical Oxidation of Histamine and Serotonin at Highly Boron-Doped Diamond Electrodes. *Anal. Chem.* **72**, 1632–1638 (2000).
34. Westerink, R. H. S. & Ewing, A. G. The PC12 cell as model for neurosecretion. *Acta Physiol (Oxf)* **192**, 273–285 (2008).

35. Melchior, J. R., Ferris, M. J., Stuber, G. D., Riddle, D. R. & Jones, S. R. Optogenetic versus electrical stimulation of dopamine terminals in the nucleus accumbens reveals local modulation of presynaptic release. *Journal of Neurochemistry* **134**, 833–844 (2015).
36. Mernier, G., Keersmaecker, K. D., Bartic, C. & Borghs, G. On-chip controlled release of neurotransmitter molecules. *Microelectronic Engineering* (2007).
37. Fabregat, G., Giménez, A., Díaz, A., Puiggalí, J. & Alemán, C. Dual-Functionalization Device for Therapy through Dopamine Release and Monitoring. *Macromolecular Bioscience* **18**, 1800014 (2018).
38. Broccard, F. D., Joshi, S., Wang, J. & Cauwenberghs, G. Neuromorphic neural interfaces: from neurophysiological inspiration to biohybrid coupling with nervous systems. *J Neural Eng* **14**, 041002 (2017).
39. Marzocchi, M. *et al.* Physical and Electrochemical Properties of PEDOT:PSS as a Tool for Controlling Cell Growth. *ACS Appl. Mater. Interfaces* **7**, 17993–18003 (2015).
40. Cellot, G. *et al.* PEDOT:PSS Interfaces Support the Development of Neuronal Synaptic Networks with Reduced Neuroglia Response In vitro. *Front Neurosci* **9**, 521 (2015).
41. Cai, X. *et al.* A D2 to D1 shift in dopaminergic inputs to midbrain 5-HT neurons causes anorexia in mice. *Nat Neurosci* **25**, 646–658 (2022).
42. Wiencke, K., Horstmann, A., Mathar, D., Villringer, A. & Neumann, J. Dopamine release, diffusion and uptake: A computational model for synaptic and volume transmission. *PLoS Comput Biol* **16**, e1008410 (2020).
43. Lee, Y., Park, H.-L., Kim, Y. & Lee, T.-W. Organic electronic synapses with low energy consumption. *Joule* **5**, 794–810 (2021).
44. Sandberg, A. Energetics of the brain and AI. (2016).
45. Keene, S. T. *et al.* A biohybrid synapse with neurotransmitter-mediated plasticity. *Nat. Mater.* **19**, 969–973 (2020).
46. Koutsouras, D. A., Torricelli, F., Gkoupidenis, P. & Blom, P. W. M. Efficient Gating of Organic Electrochemical Transistors with In-Plane Gate Electrodes. *Adv. Mater. Technol.* 2100732 (2021) doi:10.1002/admt.202100732.

47. Tony, A. *et al.* The Additive Manufacturing Approach to Polydimethylsiloxane (PDMS) Microfluidic Devices: Review and Future Directions. *Polymers* **15**, 1926 (2023).
48. Ito, H. T. Frequency-dependent signal transmission and modulation by neuromodulators. *Front. Neurosci.* **2**, 138–144 (2008).
49. Khamechian, M. B. & Daliri, M. R. Frequency modulation of cortical rhythmicity governs behavioral variability, excitability and synchrony of neurons in the visual cortex. *Sci Rep* **12**, 20914 (2022).
50. Marinelli, M. & McCutcheon, J. E. Heterogeneity of dopamine neuron activity across traits and states. *Neuroscience* **282**, 176–197 (2014).
51. Thurley, K., Senn, W. & Lüscher, H.-R. Dopamine Increases the Gain of the Input-Output Response of Rat Prefrontal Pyramidal Neurons. *Journal of Neurophysiology* **99**, 2985–2997 (2008).
52. Zhang, D.-Q. *et al.* Intraretinal signaling by ganglion cell photoreceptors to dopaminergic amacrine neurons. *Proc. Natl. Acad. Sci. U.S.A.* **105**, 14181–14186 (2008).
53. Huang, W. *et al.* Vertical organic electrochemical transistors for complementary circuits. *Nature* **613**, 496–502 (2023).
